# Trends in Musculoskeletal Rehabilitation Needs in China From 1990 to 2030: A Bayesian Age-Period-Cohort Modeling Study

**DOI:** 10.3389/fpubh.2022.869239

**Published:** 2022-06-15

**Authors:** Ningjing Chen, Daniel Yee Tak Fong, Janet Yuen Ha Wong

**Affiliations:** School of Nursing, Li Ka Shing Faculty of Medicine, The University of Hong Kong, Hong Kong SAR, China

**Keywords:** rehabilitation, musculoskeletal disorders, prevalence, years lived with disability, prediction

## Abstract

**Background:**

Disability and medical expenses caused by musculoskeletal disorders in China had a great impact on the global health and economy. Rehabilitation is essential for dealing with musculoskeletal disorders. However, China's musculoskeletal rehabilitation needs remain unknown. This study aimed to examine the secular trends for musculoskeletal rehabilitation needs in China from 1990 to 2030.

**Methods:**

Data on musculoskeletal rehabilitation needs were extracted from the Global Burden of Diseases, Injuries, and Risk Factors Study (GBD) repository. Estimated annual percentage changes (EAPCs) were calculated to reflect fluctuations in the age-standardized rates. The Bayesian age-period-cohort models were used to project rehabilitation needs.

**Results:**

The number of prevalent cases and years lived with disability (YLD) counts in need of musculoskeletal rehabilitation increased greatly in China from 1990 to 2019. There will be 465.9 million Chinese people in need of rehabilitation, with the age-standardized prevalence rate increasing to 21,151.0 [2.5–97.5% predictive interval (95% *PI*) 14,872.6–27,429.3] per 100,000 persons in 2030. Similarly, the YLD counts will increase to 40.1 million, with the age-standardized YLD rate increasing to 1,811.2 (95% *PI* 1,232.5–2,390.0) per 100,000 persons in 2030.

**Conclusions:**

Increasing trends in musculoskeletal rehabilitation needs were found from 1990 to 2019, which will be anticipated through 2030. Rehabilitation is suggested to be integrated into primary care settings.

## Introduction

According to the Global Burden of Diseases, Injuries, and Risk Factors Study (GBD) 2017, musculoskeletal disorders become one of the three leading contributors to years lived with disability (YLDs) in China (1). Musculoskeletal disorders cause physical impairment, psychological incapacity, and organizational dysfunction, which lead to productivity reduction and high medical costs, placing great health and financial burdens on individuals, communities, and societies (1, 2).

Rehabilitation is essential for relieving pain, improving physical mobility, and strengthening psychological functioning (3, 4). Moreover, rehabilitation helps to reduce health expenditure by avoiding or shortening hospital stay (5). The profound disease and economic burdens caused by musculoskeletal disorders call for appropriate rehabilitation provision. It is necessary to have a deep understanding of musculoskeletal rehabilitation needs before health service planning and targeted program interventions.

The GBD 2019 study comprehensively analyses the prevalence, incidence, and YLDs of 369 diseases and injuries in 204 countries and territories between 1990 and 2019. Standardized methods for data screening, cleaning, and generation were reported in the previous GBD studies (6–8). Briefly, the GBD project inputs data from censuses, disease registries, vital statistics, civil registration, satellite monitors, health service records, and other sources (6–8). Data bias was examined and adjusted by cross-validation using the GBD's Bayesian meta-regression tool, DisMod-MR 2.1. The estimates in the GBD project were updated annually by adding newly available data and using more appropriate methodologies (6–8).

Low back pain (International Classification of Disease [ICD]-10 code M54.3-54.5, and ICD-9 code 724) is pain referred to the low back which continues for 1 day or longer (6). Neck pain (ICD-10 code M54.2 and ICD-9 code 723.1) is pain referred to the neck that lasts for at least 1 day (6). Osteoarthritis (ICD-10 code M16-19 and ICD-9 code 715), is the most common type of arthritis, presenting as long-term inflammation, malfunction, and anatomical damages in the joints (6). Rheumatoid arthritis (ICD-10 codes M05-08 excluding M07 and ICD-9 codes 714.0–714.9) refers to a systemic autoimmune dysfunction that leads to pain, swelling, and shape changes in joints and can concur with multiple manifestations (6). For estimating rehabilitation needs, seven health conditions, namely, low back pain, neck pain, osteoarthritis, rheumatoid arthritis, fractures, amputation, and other injuries, which caused the largest number of YLDs and rehabilitation was the primary management strategy, were selected in the GBD project (6). The selection process of health conditions used a stepwise method. First, 20 health conditions that resulted in the most numbers of YLDs were identified. Second, only those conditions for which rehabilitation was the necessary and key intervention was retained. Subsequently, a primary list was reviewed and discussed by professionals invited by the World Health Organization. Lastly, the list was modified if other health conditions necessitated rehabilitation and needed to be included (6). Details on the process for estimating the rehabilitation needs were reported in a previous study (6).

To our knowledge, only one study has provided an overall view of musculoskeletal rehabilitation needs at the global and regional levels using data from the GBD study 2019 (6). Although rehabilitation needs were calculated, this study did not analyze secular changes of musculoskeletal rehabilitation needs. Therefore, the shifting patterns of musculoskeletal rehabilitation needs remain unclear. The secular trends over the past decades represent changing patterns of disease epidemiology, and the disease projection in the following decades will also be valuable for disease prevention and the design of coping strategies (9). Disability and medical expenses caused by musculoskeletal disorders in China could have a great impact on global health and economy. Therefore, this study aimed to (1) examine the secular trends for prevalence and YLDs of musculoskeletal rehabilitation needs by sex, age, and category in China from 1990 to 2019, (2) assess the associations between musculoskeletal rehabilitation needs and China's societal development, and (3) project China's musculoskeletal rehabilitation needs to 2030.

## Materials and Methods

### Study Design

This is a cross-sectional study using secondary data.

### Data Sources and Samples

In this cross-sectional study, we obtained data on prevalence, YLDs, and age-standardized prevalence and YLD rates of musculoskeletal disorders in need of rehabilitation in 19 countries of the G20 excluding the European Union from 1990 to 2019 by age, sex, year, and health condition from the GBD repository (https://vizhub.healthdata.org/rehabilitation/) (10). We included all ages of participants from both sexes. In the GBD project, the 95% uncertainty interval (*UI*) for each estimate was determined by the 25th and 975th draws in the 1,000 ordered draws, respectively. To assess the associations between musculoskeletal rehabilitation needs and societal development in China, we obtained the sociodemographic index from the GBD project. The sociodemographic index is a composite indicator, which incorporates income per head, total fertility rates younger than 25 years, and the average duration of educational attainment in individuals aged above 15 years (11). To project rehabilitation needs, we also retrieved rehabilitation need data by health condition, 5-year age groups (from 0–4 to 85 years, 18 age groups in total), and year between 1990 and 2019 from the GBD repository. The Chinese population data by 5-year age groups (from 0–4 to 85 years, 18 age groups in total), and by year between 1990 and 2030 were extracted from the United Nations Department of Economics and Social Affairs Population Dynamics (https://population.un.org/wpp/Download/Standard/Population/) (12).

### Statistical Analyses

#### Trends of Rehabilitation Needs, 1990–2019

In this study, the estimated annual percentage change (EAPC) and its 95% confidence interval (*CI*) were generated to reflect fluctuations in the age-standardized rate (9, 13). The lower bound of an EAPC greater than zero indicates that the age-standardized rate increases, whereas the upper bound of an EAPC less than zero suggests that the age-standardized rate decreases and the 95% *CI* of an EAPC including zero supports that the age-standardized rate remains unchanged during the study period (14).

#### Trends of Rehabilitation Needs, 2020–2030

We used the World (WHO 2000–2025) Standard population to standardize the prevalence and YLDs in need of rehabilitation (15). In the previous studies, the age-period-cohort (APC) model, Joinpoint regression model, smooth spline model, generalized additive model, and Poisson regression model have been widely used in disease trend analyses or projections (9, 16–18). The prevalence and YLDs data in China were divided into two parts according to their years (data in the year 1990–2013, and 2014–2019). The training data (data in the year 1990–2013) were used to train the Bayesian age-period-cohort (BAPC) model, Joinpoint regression model, smooth spline model, generalized additive model, and Poisson regression model. The predicted results were compared with those in the testing data (data in the year 2014–2019). The mean absolute percentage error (%) = $\frac{100}{n}\overset{n}{\underset{n=1}{\sum }}|\left(\frac{y_{o}-y_{p}}{y_{o}}\right)|$ was estimated to evaluate model accuracy (9), where *n, y*_*o*_, and *y*_*p*_ presented the sample size, observed values, and predicted values, respectively. As the mean absolute percentage errors for both prevalent cases and YLDs in the BAPC model were the smallest (Supplementary Figure 1) among these five models, the BAPC model was selected to project the prevalence and YLDs in need of rehabilitation through 2030.

In an APC model, for age group *i* in period *j*, the logarithm of the number of disease cases λ_*ij*_ is calculated by:


(1) $$\begin{array}{r} \log\lambda_{ij}=\mu +\alpha_{i}+\beta_{j}+\gamma_{k}, \end{array}$$

where μ, α_*i*_, β_*j*_, and γ_*k*_ refer to the intercept, age, period, and cohort effects, respectively (9, 19, 20). The cohort index *k* is calculated based on the age group index *i*, period index *j*, and the ratio of year span of each age group to period. In this study, the age index *i* ranged from 1 to *I* = 18, and the ratio of year span of each age group to period is equal to 5. Therefore, *k* = 5^*^(18−*i*)+*j*. In this study, the BAPC model with integrated nested Laplace approximations was used to predict rehabilitation needs through 2030 (20). For smoothing consideration, independent mean-zero normal distributions on the second differences of all time effects were assumed in a BAPC model (20). In particular, the age effects are defined as:


(2) $$\begin{array}{r} f\left(\alpha |k_{\alpha }\right)\propto {k_{\alpha }}^{\frac{I-2}{2}}exp\left[-\frac{k_{\alpha }}{2}\overset{I}{\underset{i=3}{\sum }}{\left(\alpha_{i}-2\alpha_{i-1}+\alpha_{i-2}\right)}^{2}\right], \end{array}$$

where *k*_α_ is the variance parameter (20). To address the possible overdispersion, for a specific age group *i*, with a *t* period(s) in the following year(s), an independent random effect *z*_*ij*_ ~ *N* (0$,\kappa_{z}^{-1}$) was added to the model (1):


(3) $$\begin{array}{r} \log\lambda_{ij+t}=\;\mu \;+\;\alpha_{i}\;+\;\beta_{j+t}\;+\;\gamma_{k+t}\;+z_{ij+t}\; \end{array}$$

(20).

In a BAPC model, the period effect at period *i* + 1 is assumed to have a distribution as follows:


(4) $$\begin{array}{r} {\beta_{j}}_{+1}|\beta_{1},...,\beta_{j},\kappa_{\beta }\sim N\left(2\beta_{j}-{\beta_{j}}_{-1}{,\kappa_{\beta }}^{-1}\right) \end{array}$$

(20).

Data analyses were conducted in RStudio Version 1.3.1093 and R packages ggplot2 (21), Rcan (22), BAPC (23), and INLA (www.r-inla.org) (24, 25). The *p*-values < 0.05 were treated as statistically significant.

## Results

### Disease Burden and Rehabilitation Needs

In China, the number of prevalent cases of musculoskeletal disorders in need of rehabilitation increased greatly from 186.0 (95% *UI* 173.7–199.2) million to 322.1 (95% *UI* 301.7–343.0) million from 1990 to 2019, with a significant increase in YLD counts from 17.6 (95% *UI* 12.5–23.3) million to 28.1 (95% *UI* 19.9–38.5) million during the same period. The age-standardized prevalence rate in China decreased from 17,966.3 (95% *UI* 16,799.9–19,142.9) per 100,000 persons to 17,225.4 (95% *UI* 16,211.4 to 18,325.4) per 100,000 persons, with an EAPC of −0.10% (95% *CI* −0.13 to −0.07%) annually. Likewise, age-standardized YLD rate in China decreased from 1707.7 (95% *UI* 1,225.3–2,269.2) per 100,000 persons to 1,496.8 (95% *UI* 1073.5–2025.7) per 100,000 persons, with an EAPC of −0.34% (95% *CI* −0.45 to −0.23%) annually (Table 1). However, there was an overall increase in the rank in terms of age-standardized prevalence rate, from last in 1990 to 18th, ahead of South Africa among 19 countries of the G20 in 2019. Similarly, in terms of age-standardized YLD rate, the rank of China stabilized at 18th, followed by South Africa during the full period (Supplementary Figures 2, 3; Supplementary Table 1).

**Table 1:** Musculoskeletal rehabilitation needs and trends in China from 1990 to 2019.

	**Prevalence**					**YLDs**				
	**1990**		**2019**		**1990–2019**	**1990**		**2019**		**1990–2019**
**Health condition**	**Number** **(95% UI)**	**ASRs per 100,000 persons** **(95% UI)**	**Number** **(95% UI)**	**ASRs per 100,000 persons** **(95% UI)**	**EAPCs** **(95% CI)**	**Number** **(95% UI)**	**ASRs per 100,000 persons** **(95% UI)**	**Number** **(95% UI)**	**ASRs per 100,000 persons** **(95% UI)**	**EAPC**s **(95% CI)**
Musculoskeletal disorders	185,964,961 (173,667,989–199,200,185)	17,966.3 (16,799.9–19,142.9)	322,124,617 (301,712,945–342,970,938)	17,225.4 (16,211.4–18,325.4)	−0.10 (−0.13 to −0.07)^*^	17,590,221 (12,527,486–23,340,204)	1,707.7 (1,225.3–2,269.2)	28,051,324 (19,918,264–38,476,078)	1,496.8 (1,073.5–2,025.7)	−0.34 (−0.45 to −0.23)^*^
Low back pain	75,298,419 (65,995,275–85,123,504)	7,245.3 (6,390.0–8,170.5)	91,339,432 (80,527,993–104,119,888)	5,134.7 (4,548.5–5,787.0)	−0.76 (−0.82 to −0.71)^*^	8,552,855.9 (6,028,307.5–11,419,160.7)	815.2 (575.4–1,094.5)	10,334,104 (7,329,879–14,004,812)	579.1 (411.6–778.1)	−0.75 (−0.92 to −0.59)^*^
Neck pain	37,850,796 (29,872,568–48,558,260)	3,528.3 (2,800.5–4,485.7)	67,966,088 (53,672,885–87,161,085)	3,572.0 (2,868.2–4,517.6)	0.06 (−0.01 to 0.13)	3,814,002.2 (2,479,006.8–5,573,876.0)	352.8 (230.0–516.8)	6,791,997 (4,412,005–9,788,049)	358.1 (234.5–515.9)	0.06 (−0.16 to 0.28)
Fractures	27,764,873 (25,621,345–29,988,579)	2,816.6 (2,621.2–3,026.7)	65,506,279 (61,016,091–69,919,174)	3,514.8 (3,283.8–3,744.4)	0.31 (0.24–0.39)^*^	1,702,825.3 (1,160,164.4–2,396,954.0)	171.2 (117.5–240.5)	3,769,530 (2,538,235–5,359,283)	203.0 (136.7–289.4)	0.06 (−0.27 to 0.38)
Other injuries	22,610,162 (20,503,030–25,547,832)	2,090.6 (1,903.1–2,345.2)	40,087,387 (36,401,161–44,972,396)	2,222.7 (2,011.1–2,509.3)	0.14 (0.04–0.23)^*^	778,538.8 (547,611.5–1,062,615.8)	71.0 (50.0–96.8)	1,081,501 (702,442–1,611,643)	60.4 (39.4–90.1)	−1.01 (−1.55 to −0.47)^*^
Osteoarthritis	33,214,647 (26,155,262–40,773,803)	3,786.1 (2,983.1–4,628.0)	85,865,300 (67,598,017–105,308,306)	4,095.5 (3,235.1–5,011.9)	0.38 (0.31–0.44)^*^	1,819,338 (903,102–3,648,574)	206.4 (103.1–414.4)	4,724,885 (2,347,243–9,536,082)	224.8 (112.4–452.3)	0.40 (0.12–0.68)^*^
Amputation	14,189,313 (13,000,833–15,496,370)	1,332.3 (1,227.7–1,450.8)	27,161,966 (25,222,179–29,533,551)	1,502.7 (1,395.5–1,627.6)	−0.03 (−0.15 to 0.08)	654,217 (471,598–867,202)	63.9 (46.5–83.8)	778,981 (518,434–1,123,445)	42.8 (28.3–61.4)	−2.39 (−2.99 to −1.79)^*^
Rheumatoid arthritis	1,460,388 (1,308,985–1,627,005)	149.6 (134.5–166.3)	3,127,658 (2,808,536–3,500,555)	157.4 (141.8–174.7)	0.35 (0.02–0.68)^*^	268,445 (185,209–364,635)	27.2 (18.9–36.8)	570,326 (394,520–772,913)	28.8 (19.9–39.0)	0.37 (−0.40 to 1.15)

* *The annual percentage change in an age-standardized rate did not include 0. UI, Uncertainty interval*.

### Rehabilitation Needs by Category

**Figure 1:**
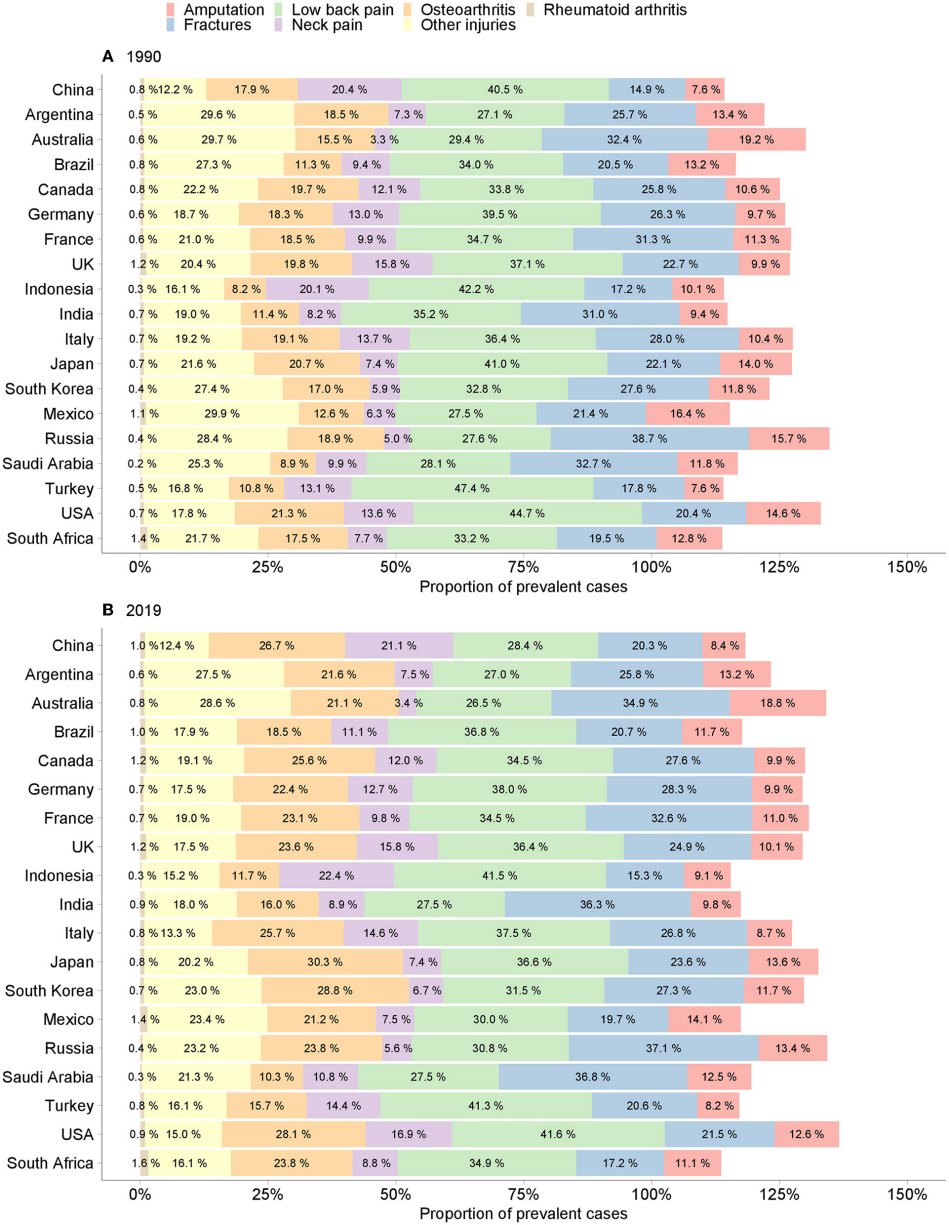
Proportion of prevalent cases of musculoskeletal rehabilitation needs in the G20 countries. **(A)** 1990. **(B)** 2019.

In both 1990 and 2019, low back pain was the major cause of total prevalent cases among the G20 countries. In 2019, the greatest contributor to total prevalent cases in China was low back pain (28.4%), followed by osteoarthritis (26.7%), neck pain (21.1%), fractures (20.3%), other injuries (12.4%), amputation (8.4%), and rheumatoid arthritis (1.0%). The proportions of prevalent cases by category increased in China, from 0.2% for rheumatoid arthritis to 8.8% for osteoarthritis, except a decrease of 12.1% for low back pain between 1990 and 2019 (Figure 1). The EAPCs increased for fractures (0.31%; 95% *CI* 0.24–0.39%), other injuries (0.14%; 95% *CI* 0.04–0.23%), osteoarthritis (0.38%; 95% *CI* 0.31–0.44%), and rheumatoid arthritis (0.35%; 95% *CI* 0.02–0.68%), remained stable for neck pain (0.06%; 95% *CI* −0.01–0.13%) and amputation (−0.03%; 95% *CI* −0.15–0.08%), and decreased for low back pain (−0.76%; 95% *CI* −0.82 to −0.71%) (Table 1).

Likewise, low back pain was also the main contributor to total YLD counts across the G20 countries. In 2019, low back pain accounted for 36.8% of the total YLD counts, followed by neck pain (24.2%), osteoarthritis (16.8%), fractures (13.4%), other injuries (3.9%), amputation (2.8%), and rheumatoid arthritis (2.0%) in China. From 1990 to 2019, the proportions of YLD counts by category in China increased by 0.5, 2.5, 3.8, and 6.5% for rheumatoid arthritis, neck pain, fractures, and osteoarthritis, but decreased by 0.6, 0.9, and 11.8% for other injuries, amputation, and low back pain, respectively (Supplementary Figure 4). The EAPCs increased for osteoarthritis (0.40%; 95% *CI* 0.12–0.68%), remained stable for neck pain (0.06%; 95% *CI* −0.16–0.28%), fractures (0.06%; 95% *CI* −0.27–0.38%), and rheumatoid arthritis (0.37%; 95% *CI* −0.40–1.15%), and decreased for low back pain (−0.75%; 95% *CI* −0.92 to −0.59%), other injuries (−1.01%; 95% *CI* −1.55 to −0.47%), and amputation (−2.39%; 95% *CI* −2.99 to −1.79%) (Table 1).

### Sex and Age Differences

During the entire study period, both age-standardized prevalence and YLD rates of low back pain, neck pain, osteoarthritis, and rheumatoid arthritis were higher in women, whereas those of fractures, other injuries, and amputation were higher in men. The age-standardized prevalence rates increased faster in women for osteoarthritis (0.49%; 95% *CI* 0.43–0.55%), and other injuries (0.18%; 95% *CI* 0.08–0.28%) but increased faster in men for fractures (0.45%; 95% *CI* 0.37–0.52%), and decreased faster in men for low back pain (−0.80%; 95% *CI* −0.86 to −0.74%) (Supplementary Figure 5). The age-standardized YLD rates decreased faster in women for amputation (−2.65%; 95% *CI* −3.31 to −1.98%), and other injuries (−1.10%; 95% *CI* −1.68 to −0.52%), but decreased faster in men for low back pain (−0.79%; 95% *CI* −0.97 to −0.62%) (Supplementary Figure 6).

Overall, musculoskeletal rehabilitation needs increased with age. Notably, sharp increasing trends in prevalence rates were found in fractures, other injuries, and amputation in people aged 90 years and over. The prevalence rates peaked at 75 years for neck pain and 75–80 years for rheumatoid arthritis in both sexes (Supplementary Figure 7). The trends in YLD rates resembled those of prevalence rates (Supplementary Figure 8).

### Associations Between Rehabilitation Needs and Sociodemographic Index

**Figure 2:**
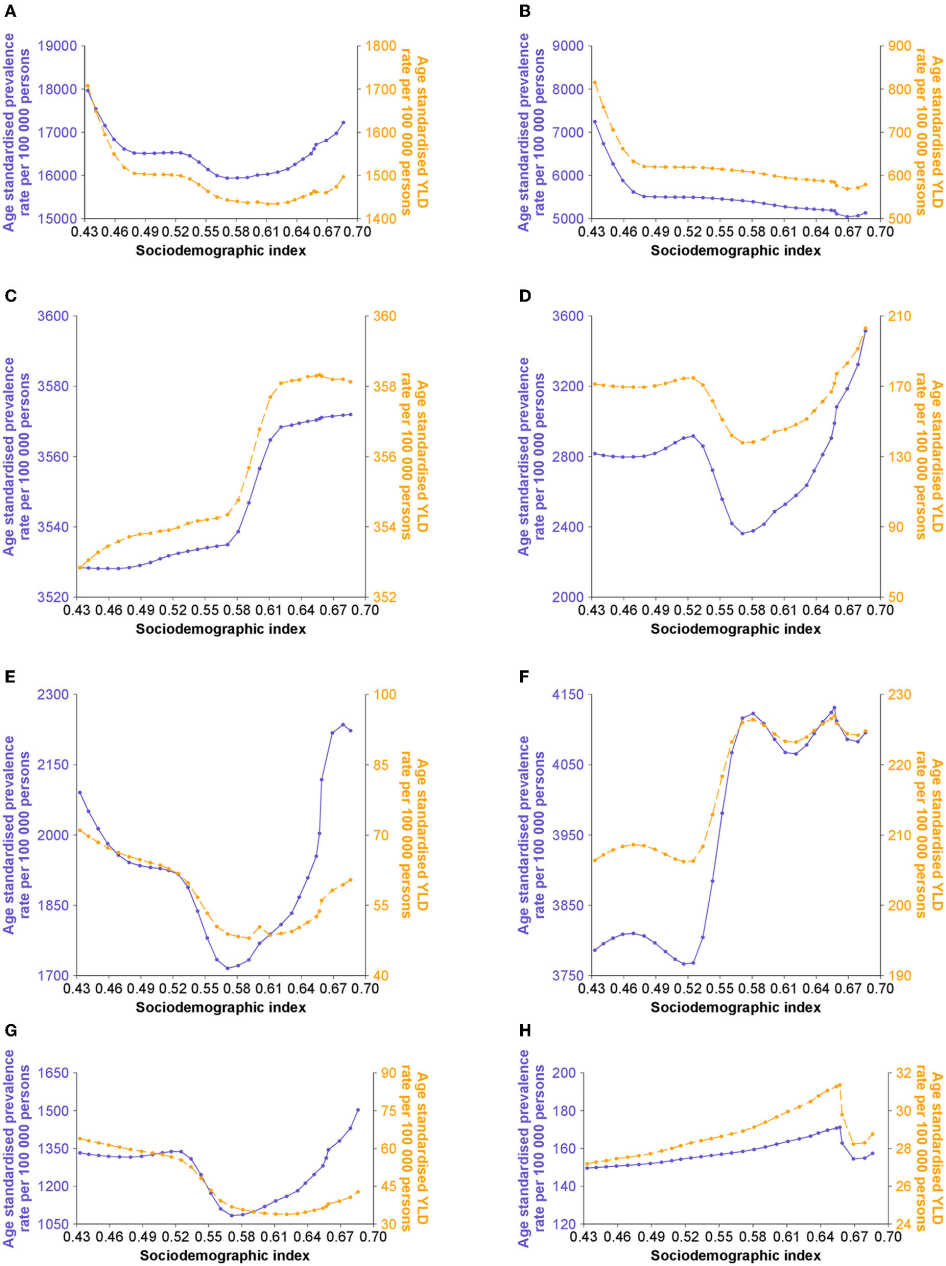
Age-standardized rates of musculoskeletal rehabilitation needs by sociodemographic index in China. **(A)** Musculoskeletal disorders. **(B)** Low back pain. **(C)** Neck pain. **(D)** Fractures. **(E)** Other injuries. **(F)** Osteoarthritis. **(G)** Amputation. **(H)** Rheumatoid arthritis.

With increasing sociodemographic index, the age-standardized prevalence rate of low back pain decreased curvilinearly, whereas that of neck pain increased curvilinearly, and that of osteoarthritis increased in a zigzag pattern. The age-standardized prevalence rate of fractures, other injuries, and amputation approached the lowest level when the sociodemographic index increased to 0.57 in 2005. However, the age-standardized prevalence rate of rheumatoid arthritis peaked when the sociodemographic index was 0.66 in 2015 (Figure 2H; Supplementary Tables 2–4). The non-linear associations between the age-standardized YLD rate and the sociodemographic index were in line with those of associations between the age-standardized prevalence rate and the sociodemographic index (Figure 2; Supplementary Tables 2–4).

### Projections to 2030

**Figure 3:**
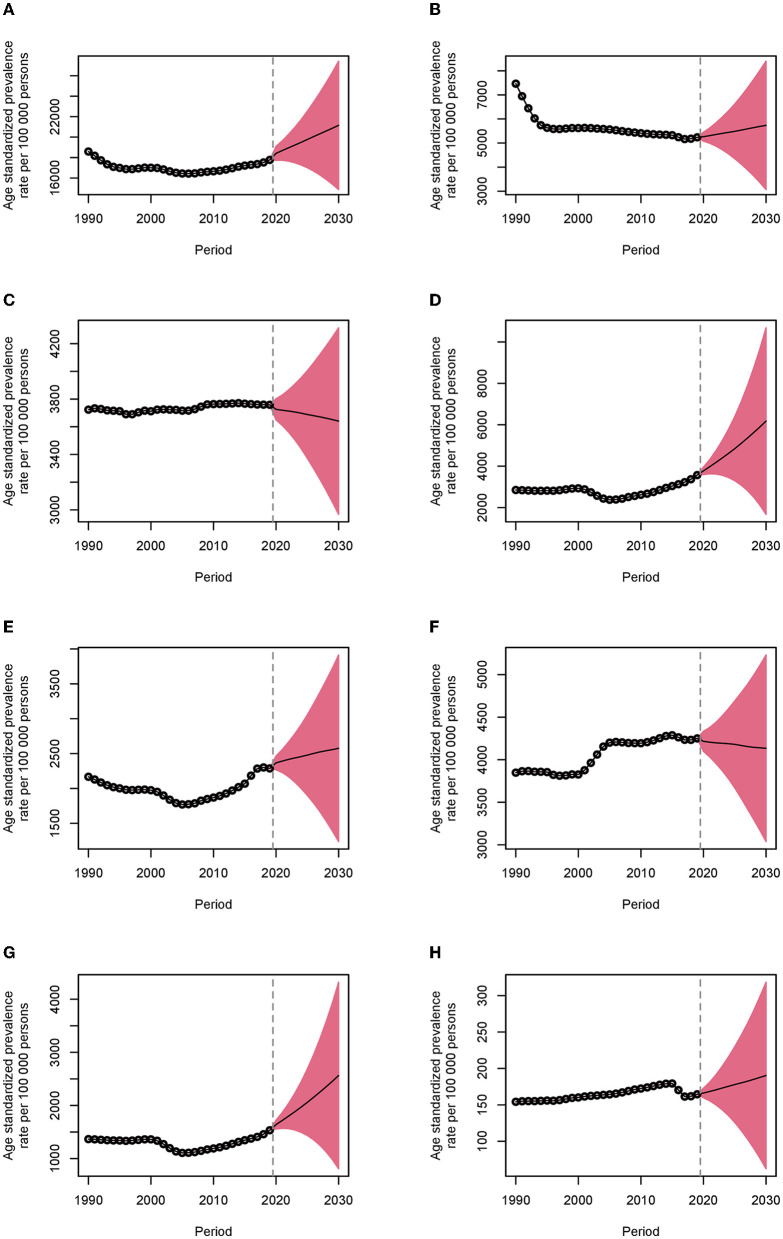
Age-standardized prevalence rates of musculoskeletal rehabilitation needs in China from 1990 to 2030. The dots indicate the observed rates, and the fan plot presents the predicted values with 2.5 and 97.5% quantiles. The solid line indicates the predicted mean values. The vertical dashed line shows when the prediction begins. **(A)** Musculoskeletal disorders. **(B)** Low back pain. **(C)** Neck pain. **(D)** Fractures. **(E)** Other injuries. **(F)** Osteoarthritis. **(G)** Amputation. **(H)** Rheumatoid arthritis.

The number of Chinese people in need of musculoskeletal rehabilitation will increase markedly from 342.0 million in 2020 to 465.9 million in 2030, with the age-standardized prevalence rate increasing from 18,410.7 [2.5–97.5%; predictive interval (95% *PI*) 17,729.7–19,091.7] per 100,000 persons in 2020 to 21,151.0 (95% *PI* 14,872.6–27,429.3) per 100,000 persons in 2030, with an EAPC of 1.39% (95% *CI* 1.26–1.53%). Similarly, the number of YLD counts will increase significantly from 29.7 million in 2020 to 40.1 million in 2030, with the age-standardized YLD rate increasing from 1,595.5 (95% *PI* 1,535.1–1,656.0) per 100,000 persons in 2020 to 1,811.2 (95% *PI* 1,232.5–2,390.0) per 100,000 persons in 2030, with an EAPC of 1.27% (95% *CI* 0.82–1.73%). Between 2020 and 2030, both age-standardized prevalence and YLD rates will increase for low back pain, fractures, and amputation, but will remain stable for neck pain, osteoarthritis, and rheumatoid arthritis (Figure 3; Supplementary Figure 9; Table 2).

**Table 2:** Musculoskeletal rehabilitation needs and trends in China from 2020 to 2030.

	**Prevalence**					**YLDs**				
	**2020**		**2030**		**2020–2030**	**2020**		**2030**		**2020–2030**
**Health condition**	**Number**	**ASRs per 100,000 persons, mean** **(95% PI)**	**Number**	**ASRs per 100,000 persons, mean** **(95% PI)**	**EAPCs** **(95% CI)**	**Number**	**ASRs per 100,000 persons, mean** **(95% PI)**	**Number**	**ASRs per 100,000 persons, mean** **(95% PI)**	**EAPCs** **(95% CI)**
Musculoskeletal disorders	341,992,073	18,410.7 (17,729.7–19,091.7)	465,882,556	21,151.0 (14,872.6–27,429.3)	1.39 (1.26–1.53)^*^	29,697,769	1,595.5 (1,535.1–1,656.0)	40,111,805	1,811.2 (1,232.5–2,390.0)	1.27 (0.82–1.73)^*^
Low back pain	93,928,096	5,262.2 (5,078.4–5,446.1)	121,364,886	5,735.4 (3,068.1–8,402.6)	0.87 (0.61–1.12)^*^	10,611,571	594.9 (574.2–615.5)	13,532,637	646.0 (348.5–943.6)	0.83 (0.08–1.59)^*^
Neck pain	68,279,122	3,725.7 (3,649.3–3,802.2)	74,429,258	3,640.8 (2,967.0–4,314.6)	−0.23 (−0.54 to 0.08)	6,820,741	373.9 (366.4–381.3)	7,359,385	364.3 (297.4–431.2)	−0.26 (−1.22 to 0.71)
Fractures	70,921,936	3,774.5 (3,595.5–3,953.5)	154,499,784	6,176.9 (1,669.4–10,684.4)	5.04 (4.76–5.32)^*^	4,077,581	218.1 (207.9–228.3)	9,090,125	366.7 (88.5–645.0)	5.32 (4.16–6.50)^*^
Other injuries	41,984,669	2,366.6 (2,267.8–2,465.5)	53,470,459	2,575.5 (1,237.6–3,913.3)	0.84 (0.47–1.22)^*^	1,125,677	63.4 (60.5–66.4)	1,682,199	77.0 (20.2–133.8)	1.95 (−0.30 to 4.25)
Osteoarthritis	87,623,502	4,216.1 (4,098.7–4,333.6)	107,304,839	4,133.8 (3,037.4–5,230.3)	−0.20 (−0.49 to 0.09)	4,826,299	232.0 (225.5–238.5)	5,897,512	227.5 (165.3–289.6)	−0.20 (−1.43 to 1.04)
Amputation	29,692,250	1,640.3 (1,556.6–1,724.0)	58,457,346	2,563.5 (807.5–4,319.5)	4.56 (4.13–4.99)^*^	831,874	45.6 (43.8–47.4)	1,718,949	73.2 (18.5–127.9)	4.83 (2.29–7.44)^*^
Rheumatoid arthritis	3,232,565	166.3 (159.8–172.8)	4,666,850	190.3 (62.3–318.4)	1.35 (−0.06 to 2.79)	591,462	30.5 (29.4–31.7)	839,765	34.8 (12.3–57.3)	1.31 (−1.95 to 4.68)

* *The annual percentage change in an age-standardized rate did not include 0. 95% PI: 2.5–97.5% predictive interval. ASRs, age-standardized rates; EAPCs, estimated annual percentage changes; UI, uncertainty interval; YLDs, years lived with disability*.

## Discussion

To our knowledge, this is the first study to present secular trends in musculoskeletal rehabilitation needs from the past to the future in China. Between 1990 and 2019, the number of prevalent cases and YLDs of musculoskeletal disorders that would benefit from rehabilitation increased remarkedly in China. Particularly, in 2019, musculoskeletal disorders contributed to 19.14% of total YLD counts (7). In addition, musculoskeletal disorders were ranked second to first among the leading 22 diseases and injuries that caused the largest number of YLD counts (1). The increasing trends were projected to continue through 2030 by using the BAPC models. This could be partly explained by population growth and aging (14). Notably, from 1990 to 2019, the number of Chinese people increased considerably from 1,183.0 to 1,441.9 million, and the number of people aged 65 and older increased greatly from 66.8 to 165.9 million, with its share more than doubling from 5.6 to 11.5% (12). In the same vein, from 2020 to 2030, the Chinese population will grow from 1,447.5 to 1,473.1 million, and the number of people aged 65 and older will increase significantly from 173.7 to 249.2 million, with its share increasing greatly from 12.0 to 16.9% (12).

Particularly, low back pain accounted for ~40% of total YLD counts due to musculoskeletal disorders in 2019. In fact, low back pain was also the primary cause of YLDs in more than 120 countries and the leading contributor to economic burdens globally (26). For example, the US government spends more than US$100 billion annually on treating patients with low back pain (27). Although there were no latest reports on the Chinese medical expenditure on low back pain, given that China has the largest number of people in the world, the medical expenses and economic loss caused by low back pain are expected to be enormous. Furthermore, there were 322.1 million Chinese people with musculoskeletal disorders who would benefit from rehabilitation in 2019, indicating that approximately one in every seven, which will increase to nearly one in every three individuals will benefit from musculoskeletal rehabilitation in 2030. This challenges the common belief that only a few people with specific disabilities need rehabilitation (6).

In terms of both age-standardized prevalence and YLD rates of musculoskeletal disorders, China was ranked from lowest to second-lowest among the G20 countries over the full study period. In addition to the largest population, this could be explained by a more remarkable improvement in life expectancy at birth in China than that in other countries. For example, from 1980 to 2019, life expectancy at birth in China increased significantly from 64.4 to 77.6 years, whereas that in the USA only increased slightly from 74.0 to 78.9 years (11). As such, it is possible that a generally higher proportion of younger people was found in China than that in other countries during the study period. As supported by the World Bank report, the share of people aged 65 and older in the total population was lower in China than that in the UK, the USA, Australia, and Russia from 1990 to 2019 (28). Therefore, age-standardized prevalence and YLD rates of musculoskeletal disorders in China tended to be lower than those in other countries since the burdens of musculoskeletal disorders increase with age (29). In fact, the disease burdens increase with age could not only be found in China but also in all other countries of the G20. Given that rehabilitation needs have increased considerably in the most recent 3 years, and the continuing increasing trends of rehabilitation needs projected by the BAPC models in the following decade, rehabilitation needs in China should not be neglected, and urgent action should be undertaken by the Chinese decision-makers and health professionals.

Sex disparities in rehabilitation needs were also observed. In this study, rehabilitation needs due to fractures, other injuries, and amputation were higher in men, whereas those of low back pain, neck pain, osteoarthritis, and rheumatoid arthritis were higher in women. This phenomenon could be partly attributed to complex traumatic, anatomic, and hormonal effects on the progression of musculoskeletal disorders. For example, men report more rehabilitation needs due to fractures, as men often suffer more serious traumas, such as vehicle accidents, vocational excessive bone loading and recreational injuries (30), although women after menopause are at a higher risk of fractures resulting from reduced bone mass, decreased estrogens, and a higher fall rate (31). However, the impact of sex differed in some countries. For example, men in Turkey and Saudi Arabia have higher rehabilitation needs due to low back pain than women, which was consistent with the previous studies (32, 33). The reason might be that more original studies included in the GBD study 2019 were conducted on the healthcare staff rather than the general population. Therefore, it should be the occupation rather than gender that affects rehabilitation needs because more men might involve in tasks, e.g., transferring patients or lifting objects requiring greater physical demands (33). In addition, men showed higher rehabilitation needs due to osteoarthritis in South Africa, which might be attributed to a higher proportion of included studies that investigated cervical and lumbar spine osteoarthritis because men had a higher risk of osteoarthritis in these sites (34).

Generally, age-standardized prevalence and YLD rates of musculoskeletal orders increased with the sociodemographic index, which was noted in all 19 countries of the G20. Over the last 30 years, rapid development has been observed in the Chinese economy (35). As reported in previous studies, risk factors for musculoskeletal orders, such as sedentary behavior, reduced physical activity, an unhealthy diet, high body mass index, smoking, and alcohol consumption are more common in people with better socioeconomic status (14, 29, 36). Particularly, a growing number of workers spend the majority of their time in the office with less body exercise, resulting in increasing burdens of low back and neck pain (37). Interestingly, an overall negative relationship was observed between age-standardized rates of low back pain and sociodemographic index, which might reveal the combined effects of the risk factors, population growth, and aging (38). Therefore, to manage the disease burdens more effectively, coping strategies should be multisectoral, including counteracting the effects of risk factors, monitoring fertility rates and live births, and tracking mortality and morbidity. Such efforts will help to promote the wellbeing of Chinese people and benefit societal development.

### Implications for Clinical Practice

High rehabilitation needs pose a major challenge to Chinese health systems. In China, the primary care settings mainly provide basic clinical treatment and public healthcare (39). However, rehabilitation is often marginalized as a natural process of recovery, rather than an essential resolution to deal with body disabilities (40). One feasible method to meet high musculoskeletal rehabilitation needs is to counter this view and integrate rehabilitation into Chinese primary care settings, in which early rehabilitation can considerably reduce the prevalence and disability of persistent musculoskeletal symptoms. For example, strength training and exercise programs for osteoarthritis can greatly improve joint stability and function (41). To provide better rehabilitation services in primary care, more professional training should be organized for health professionals, such as physicians, nurses, and community health workers to identify rehabilitation needs and deliver rehabilitation care appropriately (6), given that such training is often insufficient (39). Additionally, trained occupational and physical therapists should also be employed to better guide the implementation of interventions (6). To achieve this, the rehabilitation workforce should be given adequate remuneration and opportunities for career promotion. Furthermore, with the help of new technologies, online rehabilitation programs are encouraged to supplement the limited rehabilitation resources and improved the accessibility of rehabilitation services, especially in remote areas (42).

### Limitations

There were several limitations in this study. First, there might be an underestimation of trends in rehabilitation needs, as the understanding and diagnostic criteria for musculoskeletal disorders may have been improved overtime. However, this potential bias was adjusted for during the cause list of diseases mapped to ICD codes. Second, only seven health conditions contributing to the largest number of YLDs and necessitating rehabilitation care were included. However, other categories of musculoskeletal disorders, for example, gout, which might also need rehabilitation services, were excluded. Third, we obtained aggregate data at the national level from the GBD repository, in which data at the subnational level were not available. Therefore, we failed to compare rehabilitation needs among different provinces or between urban and rural areas, which facilitated more specific strategies for rehabilitation initiatives, given that disparities existed in medical resource allocation and health service provision across China (1, 29). Fourth, for the diagnosis of musculoskeletal disorders, although data were adjusted for case definition and outliers were removed, potential overlap might still exist in data from primary healthcare institutions or hospitals. Lastly, to achieve the goals described in the Healthy China 2030 (43), the launch of campaigns focusing on the healthy lifestyles and body fitness in the future may lead to decreases in rehabilitation needs, which may bias our predicted results. Given that we went through rigorous model selection procedures and used data from a relatively long period (1990–2019), this study not only advances our knowledge of the secular trends in musculoskeletal rehabilitation needs but also serves as a baseline for future resource distributions and disease control for China and other countries, as this is the first time to predict rehabilitation needs from the past to the future at the national level.

## Conclusions

China's musculoskeletal rehabilitation needs have increased substantially over the last three decades. There will be continuing increasing trends in musculoskeletal rehabilitation needs from 2020 to 2030. Rehabilitation services are suggested to be integrated into Chinese primary care settings.

## Data Availability Statement

The original contributions presented in the study are included in the article/Supplementary Material, further inquiries can be directed to the corresponding author.

## Ethics Statement

Ethical review and approval was not required for the study on human participants in accordance with the local legislation and institutional requirements. Written informed consent from the participants' legal guardian/next of kin was not required to participate in this study in accordance with the national legislation and the institutional requirements.

## Author Contributions

NC, DF, and JW interpreted the results. NC drafted the manuscript. DF and JW supervised the study. All authors contributed to the critical revision of the manuscript for important intellectual content and approved the final manuscript as submitted.

## Conflict of Interest

The authors declare that the research was conducted in the absence of any commercial or financial relationships that could be construed as a potential conflict of interest.

## Publisher's Note

All claims expressed in this article are solely those of the authors and do not necessarily represent those of their affiliated organizations, or those of the publisher, the editors and the reviewers. Any product that may be evaluated in this article, or claim that may be made by its manufacturer, is not guaranteed or endorsed by the publisher.

## Abbreviations

APC: age-period-cohort
BAPC: Bayesian age-period-cohort
CI: confidence interval
EAPC: estimated annual percentage change
GBD, Global Burden of Diseases, Injuries: and Risk Factors Study
ICD: International Classification of Disease
UI: uncertainty interval
YLDs: years lived with disability.
