# Supplementary material for: Trends in Musculoskeletal Rehabilitation Needs in China From 1990 to 2030: A Bayesian Age-Period-Cohort Modeling Study
Source: Front Public Health. 2022 Jun 15;10:869239. doi: 10.3389/fpubh.2022.869239 (PMC9240767; doi:10.3389/fpubh.2022.869239)
Supplement: Supplementary file 1 [file Table_1.DOCX]

Supplementary materials

Supplementary table 1. Age-standardized rates of musculoskeletal rehabilitation needs, corresponding rank in 19 countries

Supplementary table 2. Sociodemographic index, age-standardized rates of musculoskeletal rehabilitation needs in China, 1990-1999

Supplementary table 3. Sociodemographic index, age-standardized rates of musculoskeletal rehabilitation needs in China, 2000-2009

Supplementary table 4. Sociodemographic index, age-standardized rates of musculoskeletal rehabilitation needs in China, 2010-2019

Supplementary table 1. Age-standardized rates of musculoskeletal rehabilitation needs, corresponding rank in 19 countries

|  | Age-standardized prevalence rate per 100 000 persons | | | | Age-standardized YLD rate per 100 000 persons | | | |
| --- | --- | --- | --- | --- | --- | --- | --- | --- |
|  | 1990 | | 2019 | | 1990 | | 2019 | |
|  | Rate (95% UI) | Rank | Rate (95% UI) | Rank | Rate (95% UI) | Rank | Rate (95% UI) | Rank |
| China | 17966.3 (16799.9 to 19142.9) | 19 | 17225.4 (16211.4 to 18325.4) | 18 | 1707.7 (1225.3 to 2269.2) | 18 | 1496.8 (1073.5 to 2025.7) | 18 |
| Argentina | 26399.5 (25164.9 to 27685.3) | 10 | 25992.0 (24736.2 to 27267.5) | 10 | 2079.1 (1483.7 to 2781.5) | 12 | 1974.5 (1398.9 to 2667.5) | 12 |
| Australia | 34017.6 (32436.0 to 35811.6) | 1 | 33348.4 (31664.9 to 35247.8) | 1 | 2537.9 (1806.6 to 3402.9) | 4 | 2453.4 (1733.9 to 3310.2) | 2 |
| Brazil | 23108.9 (21903.3 to 24319.8) | 14 | 20756.6 (19628.6 to 21964.3) | 14 | 1875.5 (1345.7 to 2472.9) | 16 | 1760.7 (1250.3 to 2340.9) | 15 |
| Canada | 24629.2 (23708.7 to 25664.5) | 12 | 24448.2 (23175.1 to 25722.2) | 12 | 2090.1 (1504.9 to 2762.6) | 11 | 2152.8 (1528.0 to 2868.3) | 10 |
| France | 27609.8 (26298.1 to 29114.7) | 7 | 27126.2 (25739.5 to 28641.9) | 6 | 2335.6 (1675.8 to 3091.3) | 9 | 2312.5 (1643.1 to 3081.6) | 6 |
| Germany | 27245.9 (25865.1 to 28715.7) | 9 | 26914.8 (25495.3 to 28430.4) | 7 | 2399.4 (1706.7 to 3184.8) | 7 | 2357.7 (1683.1 to 3150.1) | 5 |
| UK | 28924.2 (27375.5 to 30614.7) | 5 | 27236.3 (25742.8 to 28782.6) | 4 | 2554.2 (1814.0 to 3358.4) | 3 | 2422.0 (1725.6 to 3199.1) | 4 |
| India | 20557.7 (19635.4 to 21539.4) | 17 | 19171.3 (18341.5 to 20051.2) | 17 | 1921.3 (1402.9 to 2516.6) | 15 | 1679.0 (1222.0 to 2207.6) | 16 |
| Indonesia | 20987.0 (19829.9 to 22302.5) | 15 | 19974.3 (18838.5 to 21260.0) | 16 | 1997.4 (1444.4 to 2622.6) | 13 | 1873.6 (1355.2 to 2465.6) | 13 |
| Italy | 27636.0 (26150.9 to 29289.0) | 6 | 24653.3 (23225.8 to 26196.1) | 11 | 2406.0 (1709.8 to 3206.2) | 6 | 2216.5 (1568.7 to 2958.8) | 7 |
| Japan | 27599.7 (26169.7 to 29099.3) | 8 | 26106.7 (24745.5 to 27527.8) | 9 | 2369.3 (1680.5 to 3152.7) | 8 | 2191.8 (1550.3 to 2942.2) | 9 |
| South Korea | 29051.0 (27718.2 to 30557.1) | 4 | 27151.2 (25856.0 to 28619.6) | 5 | 2445.2 (1736.5 to 3297.7) | 5 | 2208.8 (1548.6 to 2999.8) | 8 |
| Mexico | 23812.0 (22651.2 to 24983.2) | 13 | 21662.0 (20545.4 to 22798.9) | 13 | 1816.2 (1307.4 to 2402.2) | 17 | 1661.6 (1190.7 to 2220.7) | 17 |
| Russia | 34007.6 (32668.0 to 35525.6) | 2 | 29952.1 (28642.2 to 31477.7) | 2 | 2836.5 (2028.1 to 3771.0) | 2 | 2449.8 (1744.9 to 3288.5) | 3 |
| Saudi Arabia | 25486.8 (24229.5 to 26890.5) | 11 | 26268.9 (24948.7 to 27948.8) | 8 | 2127.1 (1529.1 to 2835.4) | 10 | 2106.7 (1503.4 to 2825.3) | 11 |
| Turkey | 20951.2 (19818.0 to 22142.3) | 16 | 20486.5 (19321.1 to 21749.0) | 15 | 1996.9 (1432.3 to 2630.0) | 14 | 1810.9 (1295.9 to 2386.9) | 14 |
| USA | 32127.7 (30660.3 to 33672.8) | 3 | 29675.6 (28517.8 to 30885.7) | 3 | 3026.9 (2165.1 to 4013.3) | 1 | 2856.5 (2067.5 to 3783.0) | 1 |
| South Africa | 18073.8 (17136.0 to 19088.8) | 18 | 16079.9 (15155.6 to 17041.5) | 19 | 1500.7 (1084.2 to 1995.5) | 19 | 1334.0 (955.6 to 1783.1) | 19 |

The country with the highest rehabilitation needs was ranked first. UI, uncertainty interval; YLDs, years lived with disability.

Supplementary table 2. Sociodemographic index, age-standardized rates of musculoskeletal rehabilitation needs in China, 1990-1999

|  | 1990 | 1991 | 1992 | 1993 | 1994 | 1995 | 1996 | 1997 | 1998 | 1999 |
| --- | --- | --- | --- | --- | --- | --- | --- | --- | --- | --- |
| Sociodemographic index | 0.433 | 0.441 | 0.450 | 0.459 | 0.469 | 0.479 | 0.489 | 0.499 | 0.508 | 0.516 |
| Musculoskeletal disorders |  |  |  |  |  |  |  |  |  |  |
| Prevalence rate | 17966.3 (16799.9 to 19142.9) | 17542.8 (16412.0 to 18696.0) | 17154.9 (16057.2 to 18286.2) | 16834.2 (15758.8 to 17964.5) | 16611.8 (15548.8 to 17729.9) | 16518.5 (15460.9 to 17621.8) | 16510.7 (15455.9 to 17625.7) | 16513.2 (15455.0 to 17604.3) | 16522.8 (15475.6 to 17626.4) | 16528.2 (15481.4 to 17624.1) |
| YLD rate | 1707.7 (1225.3 to 2269.2) | 1648.8 (1179.1 to 2193.9) | 1594.6 (1140.2 to 2124.0) | 1549.7 (1104.4 to 2069.1) | 1518.4 (1082.5 to 2035.6) | 1505.0 (1072.4 to 2019.1) | 1503.2 (1071.2 to 2017.2) | 1502.3 (1070.6 to 2019.0) | 1502.2 (1070.0 to 2018.4) | 1501.3 (1071.9 to 2023.5) |
| Low back pain |  |  |  |  |  |  |  |  |  |  |
| Prevalence rate | 7245.3 (6390.0 to 8170.5) | 6736.0 (5958.0 to 7592.6) | 6268.7 (5560.2 to 7062.8) | 5883.1 (5207.0 to 6606.0) | 5618.0 (4986.6 to 6306.3) | 5513.7 (4898.0 to 6192.6) | 5505.5 (4888.6 to 6186.9) | 5501.5 (4882.8 to 6183.5) | 5500.1 (4879.4 to 6183.0) | 5498.5 (4875.9 to 6183.5) |
| YLD rate | 815.2 (575.4 to 1094.5) | 758.0 (534.5 to 1013.9) | 705.6 (498.6 to 943.1) | 662.2 (468.3 to 888.0) | 632.5 (448.4 to 846.9) | 620.8 (439.9 to 830.2) | 619.9 (439.4 to 828.9) | 619.5 (439.5 to 828.8) | 619.4 (438.3 to 829.1) | 619.2 (438.9 to 827.2) |
| Neck pain |  |  |  |  |  |  |  |  |  |  |
| Prevalence rate | 3528.3 (2800.5 to 4485.7) | 3528.3 (2800.5 to 4485.9) | 3528.1 (2800.3 to 4485.8) | 3528.2 (2800.3 to 4486.2) | 3528.1 (2800.3 to 4486.0) | 3528.4 (2800.5 to 4486.5) | 3529.0 (2807.4 to 4504.5) | 3529.9 (2811.4 to 4482.9) | 3530.9 (2814.0 to 4462.7) | 3531.8 (2817.9 to 4470.0) |
| YLD rate | 352.8 (230.0 to 516.8) | 353.1 (230.5 to 514.8) | 353.3 (230.4 to 517.3) | 353.4 (231.1 to 516.9) | 353.6 (230.3 to 516.0) | 353.7 (231.0 to 516.9) | 353.8 (230.2 to 511.0) | 353.8 (231.1 to 510.6) | 353.9 (230.2 to 509.0) | 353.9 (229.5 to 510.7) |
| Fractures |  |  |  |  |  |  |  |  |  |  |
| Prevalence rate | 2816.6 (2621.2 to 3026.7) | 2806.6 (2612.2 to 3015.4) | 2800.2 (2606.6 to 3007.4) | 2797.3 (2603.1 to 3006.9) | 2798.2 (2601.7 to 3008.7) | 2801.5 (2604.7 to 3012.1) | 2817.5 (2618.8 to 3027.8) | 2845.5 (2643.5 to 3058.6) | 2878.7 (2674.4 to 3091.6) | 2905.1 (2699.4 to 3117.3) |
| YLD rate | 171.2 (117.5 to 240.5) | 170.5 (117.2 to 239.7) | 170.0 (116.3 to 239.3) | 169.6 (116.3 to 238.7) | 169.5 (115.8 to 238.8) | 169.5 (116.0 to 238.9) | 170.2 (116.6 to 239.8) | 171.6 (117.4 to 241.8) | 173.2 (118.6 to 244.1) | 174.4 (119.4 to 246.4) |
| Other injuries |  |  |  |  |  |  |  |  |  |  |
| Prevalence rate | 2090.6 (1903.1 to 2345.2) | 2050.4 (1867.8 to 2296.8) | 2013.4 (1835.5 to 2252.4) | 1981.4 (1807.4 to 2214.0) | 1957.0 (1785.9 to 2184.3) | 1941.3 (1772.6 to 2166.6) | 1934.6 (1766.9 to 2161.5) | 1930.8 (1763.4 to 2161.3) | 1928.4 (1760.7 to 2161.4) | 1924.3 (1756.1 to 2156.4) |
| YLD rate | 71.0 (50.0 to 96.8) | 69.7 (48.9 to 95.4) | 68.4 (48.2 to 93.9) | 67.3 (47.3 to 92.3) | 66.3 (46.4 to 90.9) | 65.3 (45.8 to 89.9) | 64.7 (45.3 to 89.4) | 64.1 (44.7 to 88.4) | 63.6 (44.2 to 87.8) | 62.8 (43.4 to 87.2) |
| Osteoarthritis |  |  |  |  |  |  |  |  |  |  |
| Prevalence rate | 3786.1 (2983.1 to 4628.0) | 3795.5 (2993.6 to 4640.9) | 3803.3 (3001.6 to 4647.8) | 3808.8 (3009.7 to 4654.3) | 3810.1 (3016.5 to 4655.8) | 3806.4 (3017.6 to 4651.3) | 3796.6 (3009.5 to 4634.3) | 3784.1 (3000.9 to 4620.0) | 3773.2 (2992.8 to 4601.3) | 3766.5 (2987.5 to 4581.8) |
| YLD rate | 206.4 (103.1 to 414.4) | 207.2 (104.0 to 415.7) | 207.9 (104.4 to 417.5) | 208.4 (104.6 to 418.0) | 208.6 (105.0 to 418.4) | 208.5 (105.2 to 417.8) | 208.0 (104.6 to 416.5) | 207.2 (104.3 to 415.8) | 206.6 (103.8 to 414.4) | 206.2 (103.4 to 411.5) |
| Amputation |  |  |  |  |  |  |  |  |  |  |
| Prevalence rate | 1332.3 (1227.7 to 1450.8) | 1326.6 (1222.3 to 1445.3) | 1322.1 (1216.8 to 1440.3) | 1318.5 (1211.9 to 1434.5) | 1316.3 (1211.0 to 1431.9) | 1315.4 (1211.5 to 1430.8) | 1318.5 (1214.5 to 1435.4) | 1325.4 (1220.7 to 1443.8) | 1332.7 (1227.2 to 1451.4) | 1337.7 (1231.6 to 1456.5) |
| YLD rate | 63.9 (46.5 to 83.8) | 63.0 (46.0 to 83.0) | 62.2 (45.3 to 82.0) | 61.3 (44.7 to 81.2) | 60.5 (43.8 to 80.4) | 59.6 (43.2 to 79.0) | 58.9 (42.5 to 77.9) | 58.2 (42.0 to 77.1) | 57.5 (41.7 to 76.1) | 56.6 (41.1 to 75.6) |
| Rheumatoid arthritis |  |  |  |  |  |  |  |  |  |  |
| Prevalence rate | 149.6 (134.5 to 166.3) | 149.9 (134.8 to 166.4) | 150.3 (135.3 to 166.8) | 150.7 (135.7 to 167.4) | 151.1 (136.3 to 167.8) | 151.5 (136.7 to 168.2) | 152.0 (137.2 to 168.8) | 152.7 (137.8 to 169.6) | 153.5 (138.4 to 170.4) | 154.3 (139.1 to 171.2) |
| YLD rate | 27.2 (18.9 to 36.8) | 27.3 (18.9 to 37.0) | 27.4 (18.9 to 37.1) | 27.5 (19.0 to 37.2) | 27.5 (19.0 to 37.3) | 27.6 (19.1 to 37.4) | 27.7 (19.2 to 37.7) | 27.9 (19.3 to 37.7) | 28.0 (19.3 to 37.9) | 28.1 (19.3 to 38.1) |

YLDs, years lived with disability.

Supplementary table 3. Sociodemographic index, age-standardized rates of musculoskeletal rehabilitation needs in China, 2000-2009

|  | 2000 | 2001 | 2002 | 2003 | 2004 | 2005 | 2006 | 2007 | 2008 | 2009 |
| --- | --- | --- | --- | --- | --- | --- | --- | --- | --- | --- |
| Sociodemographic index | 0.525 | 0.534 | 0.543 | 0.552 | 0.561 | 0.571 | 0.581 | 0.591 | 0.601 | 0.611 |
| Musculoskeletal disorders |  |  |  |  |  |  |  |  |  |  |
| Prevalence rate | 16524.0 (15472.1 to 17596.2) | 16455.6 (15404.3 to 17531.8) | 16308.8 (15248.5 to 17397.6) | 16137.9 (15068.4 to 17235.4) | 15996.5 (14923.9 to 17100.0) | 15939.0 (14862.3 to 17052.0) | 15943.0 (14877.9 to 17059.6) | 15952.3 (14909.9 to 17064.4) | 16009.3 (14967.6 to 17109.3) | 16030.0 (15007.7 to 17133.0) |
| YLD rate | 1499.4 (1071.1 to 2018.4) | 1492.1 (1065.8 to 2009.1) | 1478.7 (1055.6 to 1991.6) | 1463.4 (1042.5 to 1970.8) | 1450.2 (1032.0 to 1953.4) | 1443.2 (1026.2 to 1946.2) | 1440.1 (1023.3 to 1943.8) | 1436.8 (1020.9 to 1942.6) | 1438.5 (1023.1 to 1949.3) | 1434.0 (1018.5 to 1947.2) |
| Low back pain |  |  |  |  |  |  |  |  |  |  |
| Prevalence rate | 5495.4 (4869.5 to 6181.0) | 5487.0 (4861.8 to 6165.9) | 5473.0 (4848.9 to 6150.9) | 5455.6 (4833.3 to 6127.9) | 5436.4 (4816.3 to 6108.6) | 5417.9 (4803.8 to 6087.1) | 5391.5 (4779.4 to 6054.3) | 5353.6 (4744.9 to 6011.3) | 5312.2 (4707.7 to 5970.2) | 5274.9 (4675.0 to 5934.3) |
| YLD rate | 618.9 (437.9 to 828.6) | 618.1 (437.4 to 827.3) | 616.6 (435.9 to 825.3) | 614.6 (435.1 to 824.2) | 612.4 (433.7 to 821.5) | 610.3 (431.4 to 819.0) | 607.5 (429.9 to 812.0) | 603.5 (427.8 to 808.4) | 599.0 (423.8 to 801.7) | 595.0 (421.5 to 795.8) |
| Neck pain |  |  |  |  |  |  |  |  |  |  |
| Prevalence rate | 3532.5 (2820.4 to 4470.2) | 3533.1 (2820.7 to 4471.0) | 3533.6 (2821.0 to 4471.7) | 3534.1 (2821.3 to 4472.5) | 3534.5 (2821.6 to 4473.0) | 3534.9 (2821.9 to 4473.6) | 3538.6 (2821.2 to 4482.3) | 3546.8 (2849.3 to 4508.7) | 3556.5 (2869.3 to 4511.9) | 3564.7 (2870.6 to 4527.7) |
| YLD rate | 354.0 (229.0 to 509.0) | 354.1 (228.6 to 508.6) | 354.2 (229.0 to 509.3) | 354.2 (229.3 to 509.2) | 354.2 (228.4 to 509.1) | 354.3 (228.9 to 509.0) | 354.8 (229.2 to 509.8) | 355.7 (230.5 to 510.1) | 356.8 (232.9 to 512.2) | 357.7 (233.5 to 515.6) |
| Fractures |  |  |  |  |  |  |  |  |  |  |
| Prevalence rate | 2916.4 (2710.4 to 3126.7) | 2859.6 (2661.3 to 3061.5) | 2722.1 (2542.3 to 2907.8) | 2557.2 (2397.5 to 2723.1) | 2419.1 (2277.0 to 2565.5) | 2361.6 (2223.3 to 2499.2) | 2377.2 (2237.0 to 2512.5) | 2414.8 (2272.7 to 2549.1) | 2487.2 (2343.7 to 2626.6) | 2528.2 (2381.4 to 2666.4) |
| YLD rate | 174.7 (119.2 to 246.5) | 170.7 (116.3 to 241.1) | 161.7 (110.5 to 227.4) | 151.0 (102.8 to 211.7) | 142.0 (96.7 to 199.2) | 137.9 (93.7 to 193.7) | 138.3 (93.7 to 193.8) | 139.9 (94.6 to 196.3) | 144.1 (97.6 to 201.7) | 145.5 (98.1 to 204.7) |
| Other injuries |  |  |  |  |  |  |  |  |  |  |
| Prevalence rate | 1916.2 (1748.6 to 2146.6) | 1888.5 (1723.4 to 2116.9) | 1837.6 (1677.5 to 2062.0) | 1780.4 (1625.7 to 1995.4) | 1734.0 (1581.1 to 1941.1) | 1715.5 (1563.3 to 1922.9) | 1721.2 (1567.6 to 1930.0) | 1733.6 (1577.8 to 1942.9) | 1768.9 (1606.7 to 1979.9) | 1788.2 (1623.1 to 2002.6) |
| YLD rate | 61.8 (42.5 to 86.2) | 59.8 (40.6 to 84.0) | 56.7 (38.2 to 80.5) | 53.3 (35.9 to 76.5) | 50.5 (33.5 to 72.4) | 48.9 (32.3 to 70.9) | 48.3 (31.8 to 70.4) | 48.0 (31.2 to 70.7) | 50.4 (32.8 to 73.3) | 48.6 (31.8 to 71.9) |
| Osteoarthritis |  |  |  |  |  |  |  |  |  |  |
| Prevalence rate | 3768.0 (2989.0 to 4576.3) | 3804.4 (3015.6 to 4622.5) | 3884.3 (3077.0 to 4723.6) | 3980.9 (3155.2 to 4839.4) | 4066.9 (3229.0 to 4941.8) | 4116.2 (3268.3 to 5002.6) | 4122.8 (3276.8 to 5002.0) | 4108.5 (3263.7 to 4977.0) | 4086.0 (3239.8 to 4942.2) | 4067.3 (3218.3 to 4907.6) |
| YLD rate | 206.3 (103.5 to 412.7) | 208.4 (104.3 to 417.3) | 212.9 (106.6 to 426.5) | 218.3 (109.4 to 437.4) | 223.2 (111.7 to 446.7) | 226.0 (113.0 to 450.3) | 226.4 (113.6 to 452.9) | 225.6 (113.1 to 452.9) | 224.4 (112.5 to 450.5) | 223.3 (111.9 to 449.9) |
| Amputation |  |  |  |  |  |  |  |  |  |  |
| Prevalence rate | 1337.4 (1232.2 to 1455.8) | 1308.6 (1208.6 to 1423.5) | 1245.8 (1155.1 to 1351.8) | 1171.8 (1090.5 to 1267.4) | 1110.0 (1037.2 to 1197.8) | 1083.4 (1014.2 to 1168.7) | 1087.0 (1018.4 to 1170.4) | 1098.6 (1028.9 to 1183.2) | 1119.1 (1047.3 to 1204.5) | 1141.7 (1068.6 to 1228.9) |
| YLD rate | 55.4 (40.1 to 74.1) | 52.7 (38.2 to 71.0) | 48.2 (34.9 to 65.2) | 43.4 (31.1 to 59.0) | 39.2 (27.9 to 53.4) | 36.8 (26.0 to 50.3) | 35.7 (24.9 to 49.6) | 34.8 (24.2 to 48.6) | 34.2 (23.8 to 48.3) | 34.0 (23.5 to 48.2) |
| Rheumatoid arthritis |  |  |  |  |  |  |  |  |  |  |
| Prevalence rate | 155.0 (139.7 to 172.2) | 155.6 (140.3 to 172.9) | 156.3 (140.9 to 173.8) | 156.9 (141.5 to 174.4) | 157.6 (142.2 to 175.4) | 158.4 (142.8 to 176.4) | 159.4 (143.7 to 177.6) | 160.8 (145.0 to 179.1) | 162.2 (146.3 to 180.7) | 163.7 (147.5 to 182.2) |
| YLD rate | 28.2 (19.5 to 38.4) | 28.3 (19.7 to 38.1) | 28.3 (19.5 to 38.3) | 28.4 (19.7 to 38.5) | 28.5 (19.7 to 38.6) | 28.6 (19.9 to 38.8) | 28.8 (19.9 to 39.0) | 28.8 (19.9 to 38.9) | 28.9 (20.1 to 39.1) | 29.1 (20.2 to 39.4) |

YLDs, years lived with disability.

Supplementary table 4. Sociodemographic index, age-standardized rates of musculoskeletal rehabilitation needs in China, 2010-2019

|  | 2010 | 2011 | 2012 | 2013 | 2014 | 2015 | 2016 | 2017 | 2018 | 2019 |
| --- | --- | --- | --- | --- | --- | --- | --- | --- | --- | --- |
| Sociodemographic index | 0.621 | 0.631 | 0.638 | 0.646 | 0.654 | 0.657 | 0.659 | 0.669 | 0.679 | 0.686 |
| Musculoskeletal disorders |  |  |  |  |  |  |  |  |  |  |
| Prevalence rate | 16076.6 (15045.7 to 17168.8) | 16149.8 (15127.3 to 17238.9) | 16256.0 (15241.5 to 17342.6) | 16379.0 (15371.7 to 17463.6) | 16503.6 (15503.2 to 17586.4) | 16615.2 (15610.0 to 17696.0) | 16715.5 (15718.6 to 17795.4) | 16809.6 (15807.2 to 17887.6) | 16975.2 (15952.0 to 18061.7) | 17225.4 (16211.4 to 18325.4) |
| YLD rate | 1434.7 (1018.0 to 1949.3) | 1437.9 (1020.0 to 1953.9) | 1443.7 (1025.2 to 1966.6) | 1450.7 (1031.3 to 1976.4) | 1457.7 (1037.8 to 1985.7) | 1463.6 (1041.9 to 1994.7) | 1461.7 (1041.4 to 1990.2) | 1460.3 (1039.6 to 1986.9) | 1473.7 (1054.0 to 2005.1) | 1496.8 (1073.5 to 2025.7) |
| Low back pain |  |  |  |  |  |  |  |  |  |  |
| Prevalence rate | 5249.7 (4656.5 to 5901.4) | 5234.1 (4638.6 to 5882.6) | 5220.2 (4628.4 to 5864.3) | 5207.3 (4612.8 to 5847.1) | 5194.5 (4595.0 to 5829.9) | 5181.6 (4577.0 to 5816.0) | 5112.4 (4525.4 to 5742.2) | 5045.6 (4463.6 to 5663.8) | 5068.3 (4480.0 to 5699.1) | 5134.7 (4548.5 to 5787.0) |
| YLD rate | 592.2 (420.1 to 793.3) | 590.5 (417.8 to 790.3) | 588.9 (417.0 to 789.2) | 587.4 (416.1 to 787.9) | 586.0 (414.8 to 786.1) | 584.4 (413.5 to 783.6) | 576.6 (408.7 to 772.6) | 569.1 (402.0 to 761.8) | 571.6 (405.5 to 766.0) | 579.1 (411.6 to 778.1) |
| Neck pain |  |  |  |  |  |  |  |  |  |  |
| Prevalence rate | 3568.4 (2864.9 to 4513.1) | 3568.9 (2865.3 to 4513.9) | 3569.5 (2865.8 to 4514.7) | 3570.0 (2866.2 to 4515.5) | 3570.4 (2866.7 to 4516.2) | 3570.7 (2867.0 to 4516.6) | 3571.1 (2867.4 to 4516.9) | 3571.5 (2867.7 to 4517.2) | 3571.8 (2868.0 to 4517.5) | 3572.0 (2868.2 to 4517.6) |
| YLD rate | 358.1 (234.1 to 517.3) | 358.2 (234.0 to 516.5) | 358.2 (234.2 to 516.0) | 358.3 (234.4 to 516.4) | 358.3 (234.0 to 516.2) | 358.3 (234.5 to 516.5) | 358.3 (234.6 to 516.6) | 358.2 (233.9 to 516.2) | 358.2 (234.3 to 515.6) | 358.1 (234.5 to 515.9) |
| Fractures |  |  |  |  |  |  |  |  |  |  |
| Prevalence rate | 2578.6 (2427.9 to 2720.7) | 2636.6 (2481.5 to 2780.8) | 2718.0 (2557.7 to 2866.1) | 2810.6 (2644.6 to 2966.2) | 2904.6 (2731.8 to 3068.3) | 2989.3 (2809.2 to 3157.6) | 3082.5 (2893.2 to 3262.4) | 3184.8 (2988.1 to 3379.5) | 3323.7 (3110.7 to 3532.1) | 3514.8 (3283.8 to 3744.4) |
| YLD rate | 148.2 (100.2 to 209.2) | 151.3 (102.1 to 213.3) | 156.0 (105.1 to 220.7) | 161.3 (108.9 to 227.7) | 166.8 (112.7 to 235.5) | 171.7 (115.9 to 243.1) | 177.1 (119.3 to 251.5) | 183.1 (123.4 to 259.8) | 191.4 (128.9 to 271.9) | 203.0 (136.7 to 289.4) |
| Other injuries |  |  |  |  |  |  |  |  |  |  |
| Prevalence rate | 1808.8 (1641.1 to 2025.6) | 1833.1 (1663.0 to 2055.0) | 1867.2 (1693.7 to 2095.7) | 1908.4 (1730.8 to 2145.1) | 1954.7 (1772.2 to 2202.2) | 2003.4 (1815.5 to 2261.6) | 2117.9 (1910.4 to 2394.8) | 2217.8 (1998.2 to 2510.7) | 2235.4 (2015.4 to 2527.2) | 2222.7 (2011.1 to 2509.3) |
| YLD rate | 49.0 (32.1 to 72.6) | 49.4 (32.1 to 73.4) | 50.3 (32.8 to 74.9) | 51.4 (33.6 to 76.7) | 52.6 (34.3 to 78.5) | 53.7 (35.0 to 80.4) | 56.0 (36.4 to 83.7) | 58.2 (37.7 to 87.1) | 59.4 (38.5 to 88.9) | 60.4 (39.4 to 90.1) |
| Osteoarthritis |  |  |  |  |  |  |  |  |  |  |
| Prevalence rate | 4065.3 (3209.9 to 4893.4) | 4078.0 (3223.0 to 4909.1) | 4094.2 (3230.1 to 4929.0) | 4110.9 (3240.5 to 4950.1) | 4124.1 (3252.4 to 4966.3) | 4131.2 (3259.2 to 4974.8) | 4112.0 (3236.5 to 4959.7) | 4086.1 (3208.2 to 4932.5) | 4082.8 (3225.8 to 4961.3) | 4095.5 (3235.1 to 5011.9) |
| YLD rate | 223.2 (112.0 to 450.5) | 223.9 (112.6 to 451.8) | 224.9 (113.1 to 453.4) | 225.8 (113.4 to 455.3) | 226.5 (114.0 to 456.0) | 227.0 (114.1 to 457.3) | 225.9 (113.7 to 454.6) | 224.4 (112.2 to 450.3) | 224.2 (112.8 to 451.6) | 224.8 (112.4 to 452.3) |
| Amputation |  |  |  |  |  |  |  |  |  |  |
| Prevalence rate | 1159.6 (1084.8 to 1248.4) | 1181.9 (1104.7 to 1271.4) | 1212.0 (1132.6 to 1303.9) | 1246.2 (1163.7 to 1340.5) | 1280.8 (1194.2 to 1378.3) | 1311.8 (1223.0 to 1412.3) | 1344.6 (1252.4 to 1449.5) | 1379.4 (1284.0 to 1487.3) | 1429.7 (1328.6 to 1545.6) | 1502.7 (1395.5 to 1627.6) |
| YLD rate | 33.8 (23.0 to 48.3) | 34.1 (23.1 to 48.8) | 34.7 (23.4 to 49.6) | 35.4 (23.9 to 50.8) | 36.3 (24.3 to 52.0) | 37.0 (24.7 to 53.2) | 38.0 (25.1 to 54.6) | 39.1 (25.8 to 56.3) | 40.6 (26.9 to 58.5) | 42.8 (28.3 to 61.4) |
| Rheumatoid arthritis |  |  |  |  |  |  |  |  |  |  |
| Prevalence rate | 165.0 (148.8 to 183.6) | 166.5 (150.1 to 185.1) | 168.1 (151.6 to 186.7) | 169.6 (153.0 to 188.3) | 170.7 (154.0 to 189.5) | 171.2 (154.4 to 190.1) | 162.8 (146.5 to 181.0) | 154.5 (138.9 to 171.9) | 154.9 (139.4 to 172.3) | 157.4 (141.8 to 174.7) |
| YLD rate | 29.4 (20.4 to 39.7) | 29.7 (20.6 to 40.4) | 29.8 (20.6 to 40.5) | 30.0 (20.6 to 40.7) | 30.2 (20.8 to 41.1) | 30.5 (21.0 to 41.3) | 30.8 (21.2 to 41.8) | 31.1 (21.5 to 42.2) | 31.3 (21.6 to 42.4) | 31.4 (21.8 to 42.6) |

YLDs, years lived with disability.

Supplementary figure 1. The results of five model comparison based on data of China

Supplementary figure 2. Age-standardized rates of musculoskeletal rehabilitation needs in the G20 countries

Supplementary figure 3. Rank of age-standardized rates of musculoskeletal rehabilitation needs of China in the G20 countries

Supplementary figure 4. Proportion of YLD counts of musculoskeletal rehabilitation needs in the G20 countries

Supplementary figure 5. Age-standardized prevalence rate of musculoskeletal rehabilitation needs in China

Supplementary figure 6. Age-standardized YLD rate of musculoskeletal rehabilitation needs in China

Supplementary figure 7. Prevalence rate of musculoskeletal rehabilitation needs by age in China, 2019

Note: Dashed lines show 95% uncertainty intervals.

Supplementary figure 8. YLD rate of musculoskeletal rehabilitation needs by age in China, 2019

Supplementary figure 9. Age-standardized YLD rate of musculoskeletal rehabilitation needs in China from 1990 to 2030

Note: The dots indicate the observed rates, and the fan plot presents the predicted values with 2.5 and 97.5% quantiles. The solid line indicates the predicted mean values. The vertical dashed line shows when the prediction begins.
