# Supplementary figures and images for: Trends in Musculoskeletal Rehabilitation Needs in China From 1990 to 2030: A Bayesian Age-Period-Cohort Modeling Study
Source: Front Public Health. 2022 Jun 15;10:869239. doi: 10.3389/fpubh.2022.869239 (PMC9240767; doi:10.3389/fpubh.2022.869239)

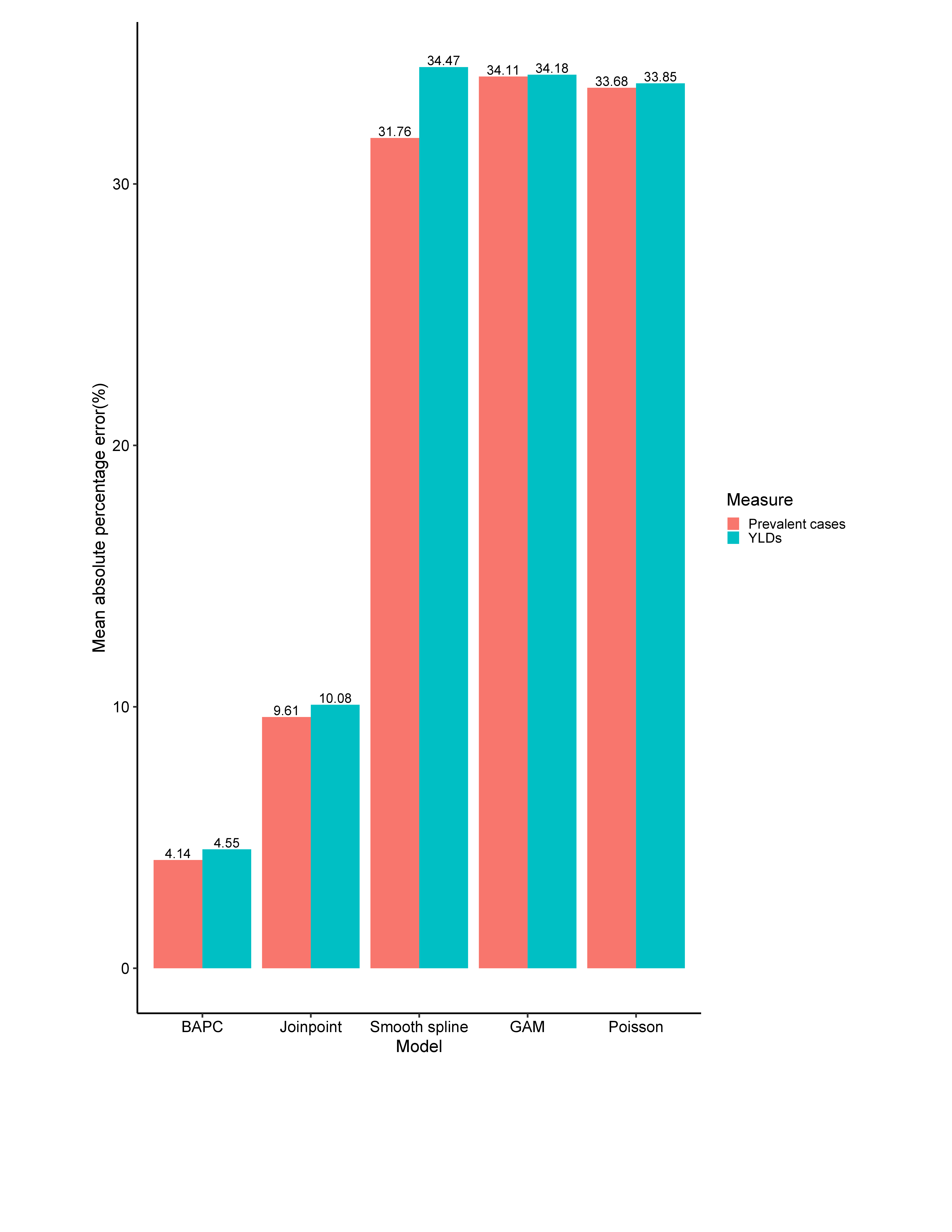

Supplement: Supplementary file 2 [file Data_Sheet_1.zip › Supplementary Figure 1.TIFF]

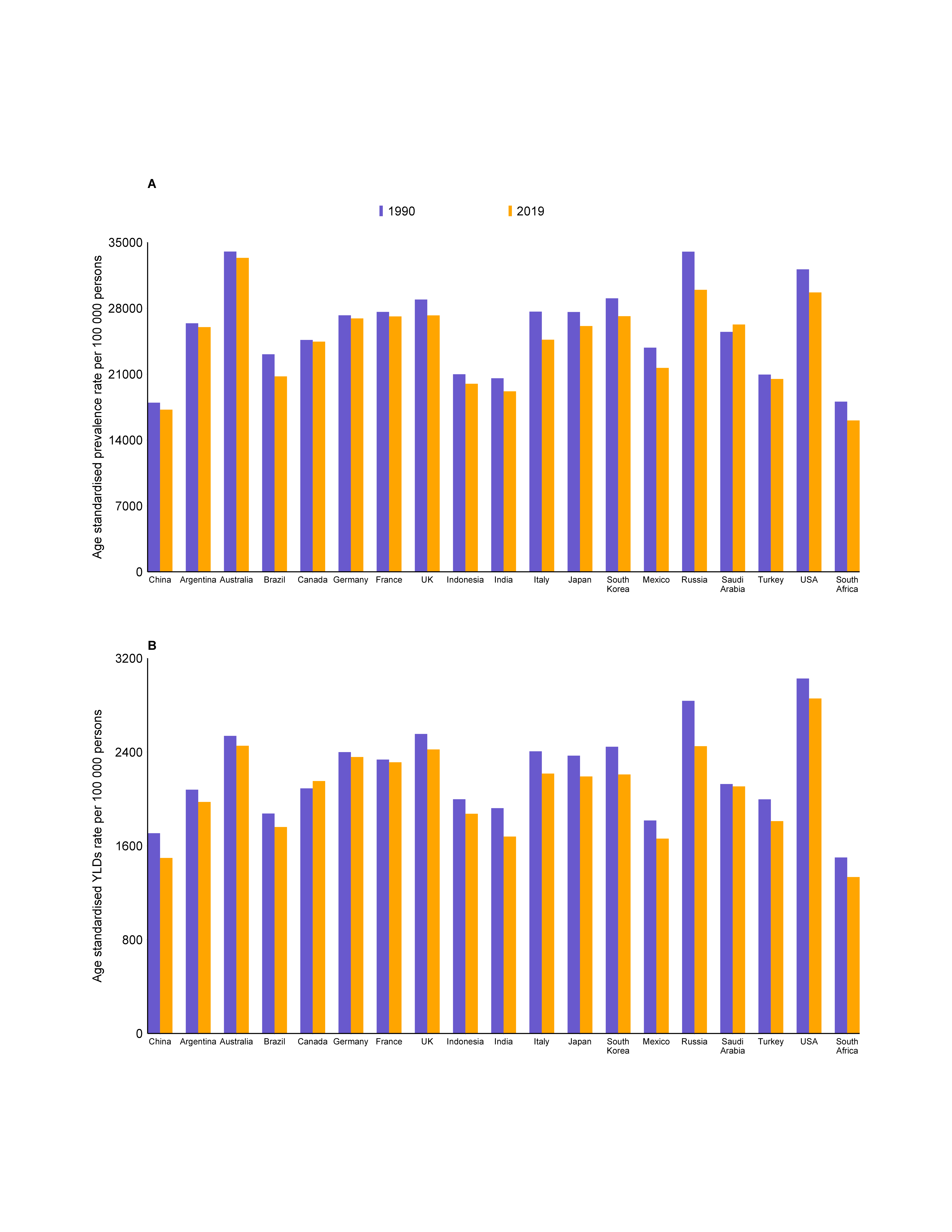

Supplement: Supplementary file 2 [file Data_Sheet_1.zip › Supplementary Figure 2.TIFF]

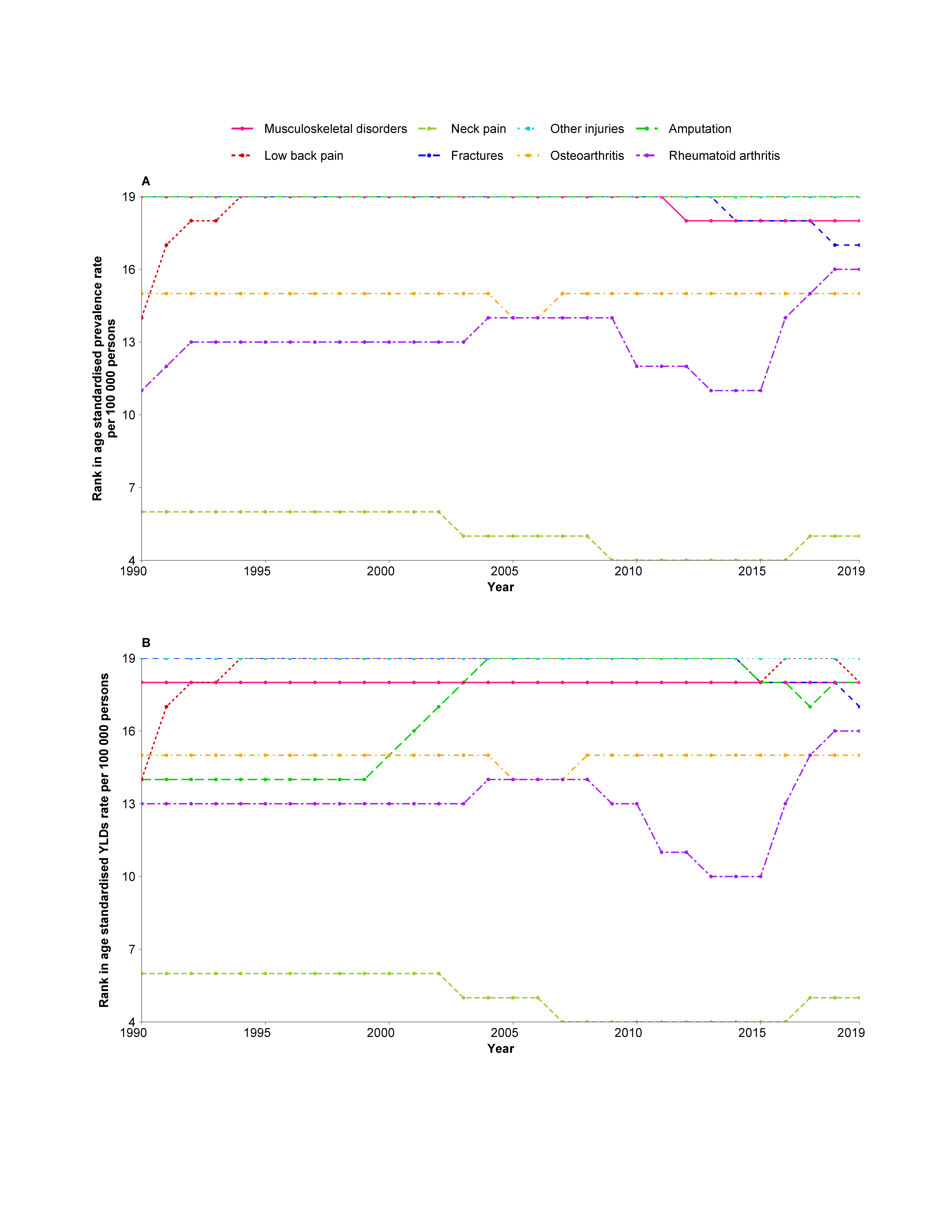

Supplement: Supplementary file 2 [file Data_Sheet_1.zip › Supplementary Figure 3.TIFF]

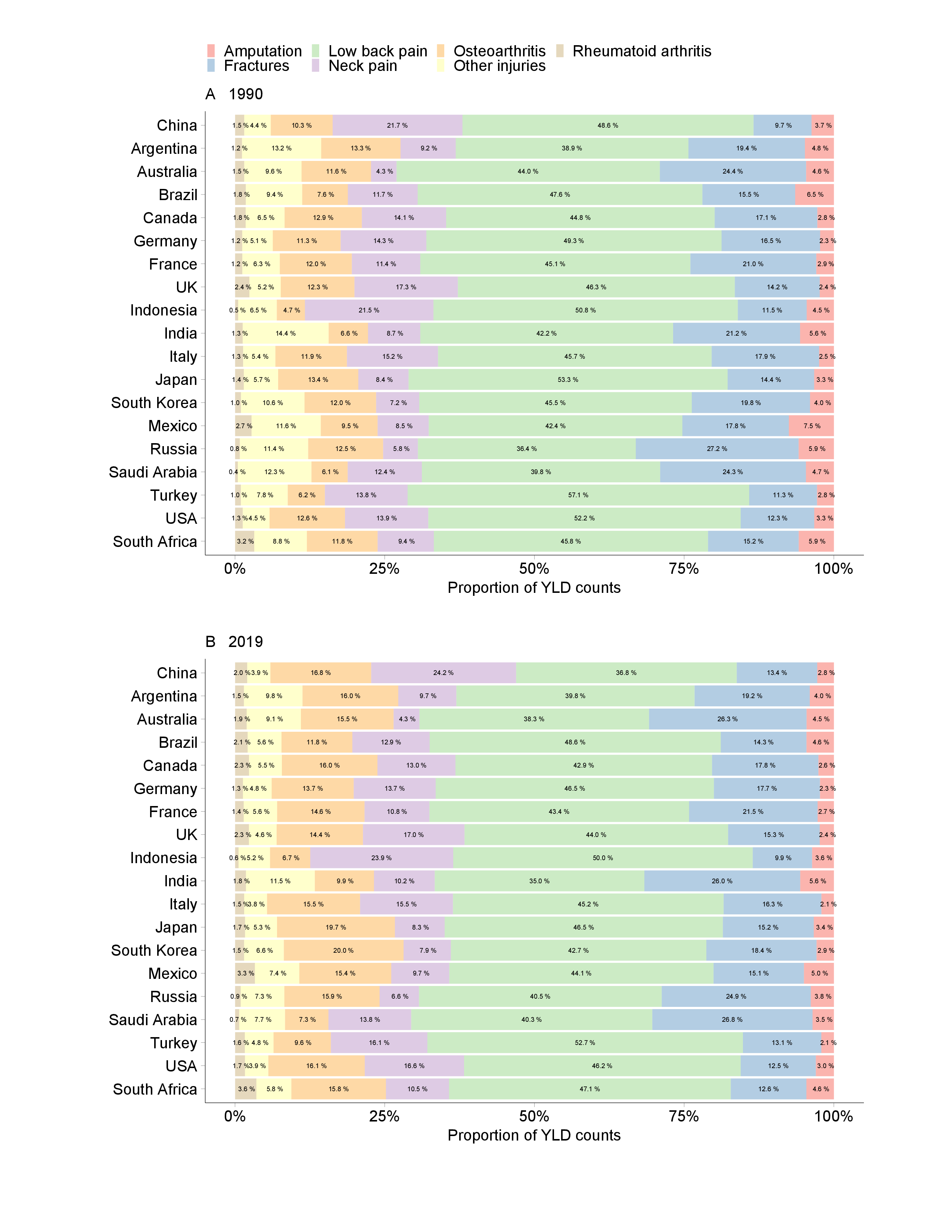

Supplement: Supplementary file 2 [file Data_Sheet_1.zip › Supplementary Figure 4.TIFF]

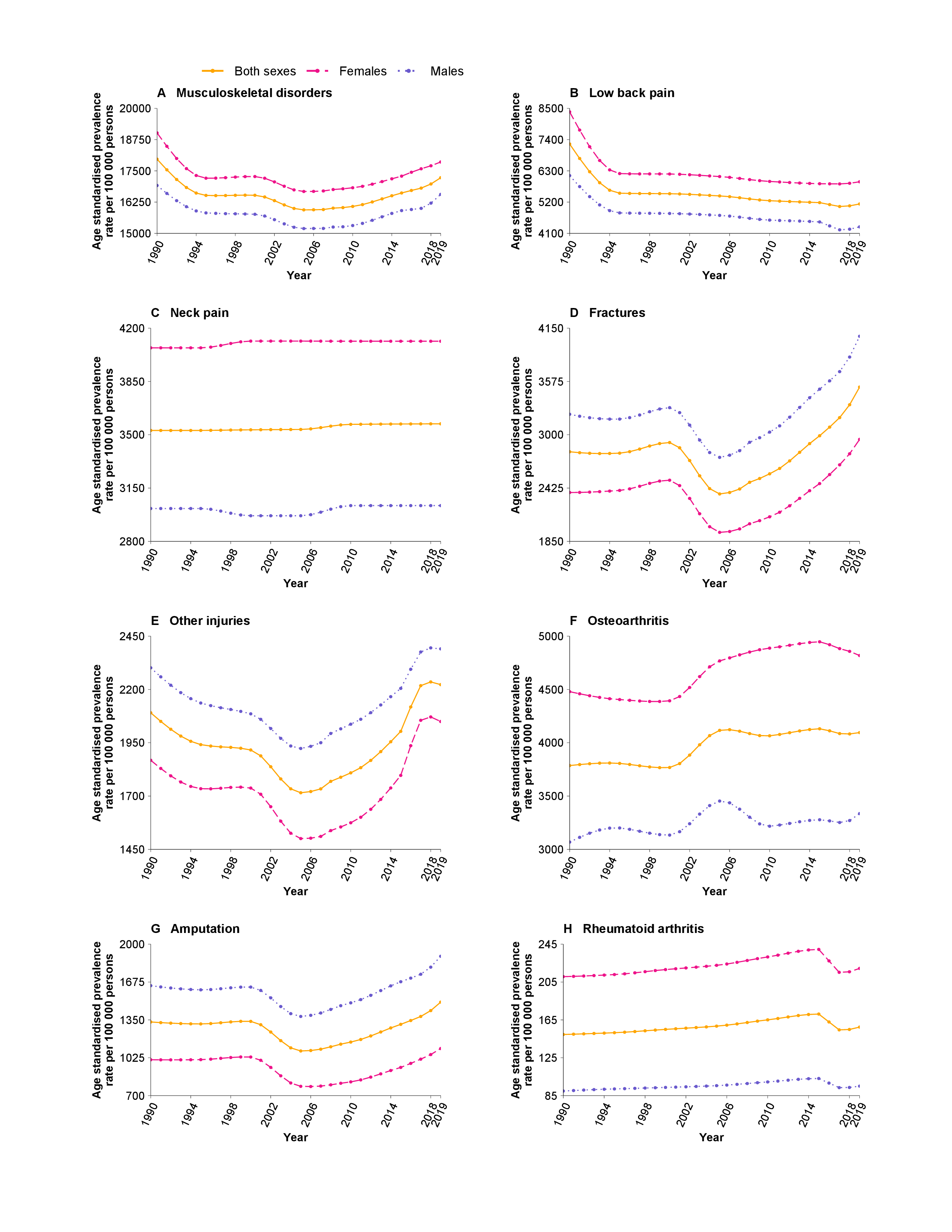

Supplement: Supplementary file 2 [file Data_Sheet_1.zip › Supplementary Figure 5.TIFF]

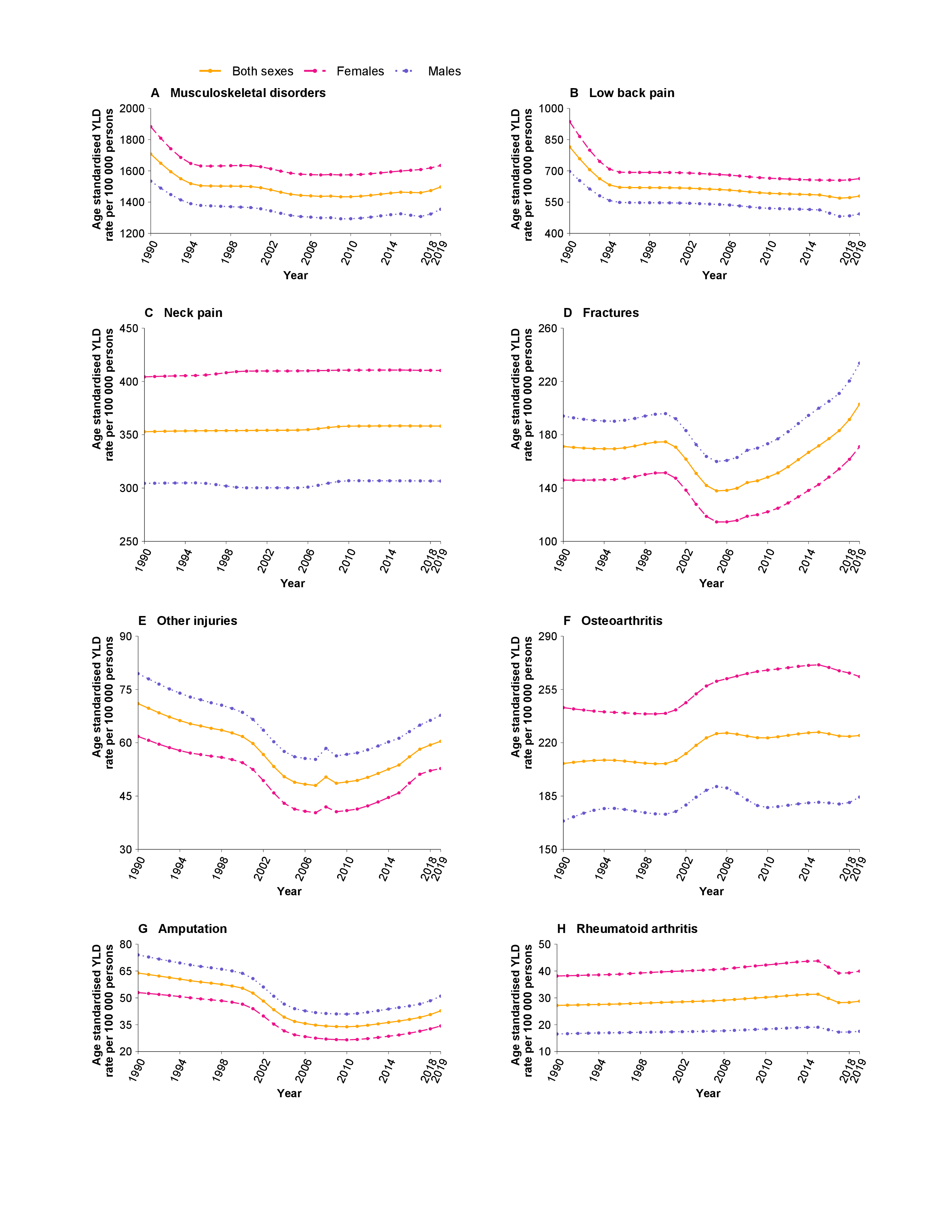

Supplement: Supplementary file 2 [file Data_Sheet_1.zip › Supplementary Figure 6.TIFF]

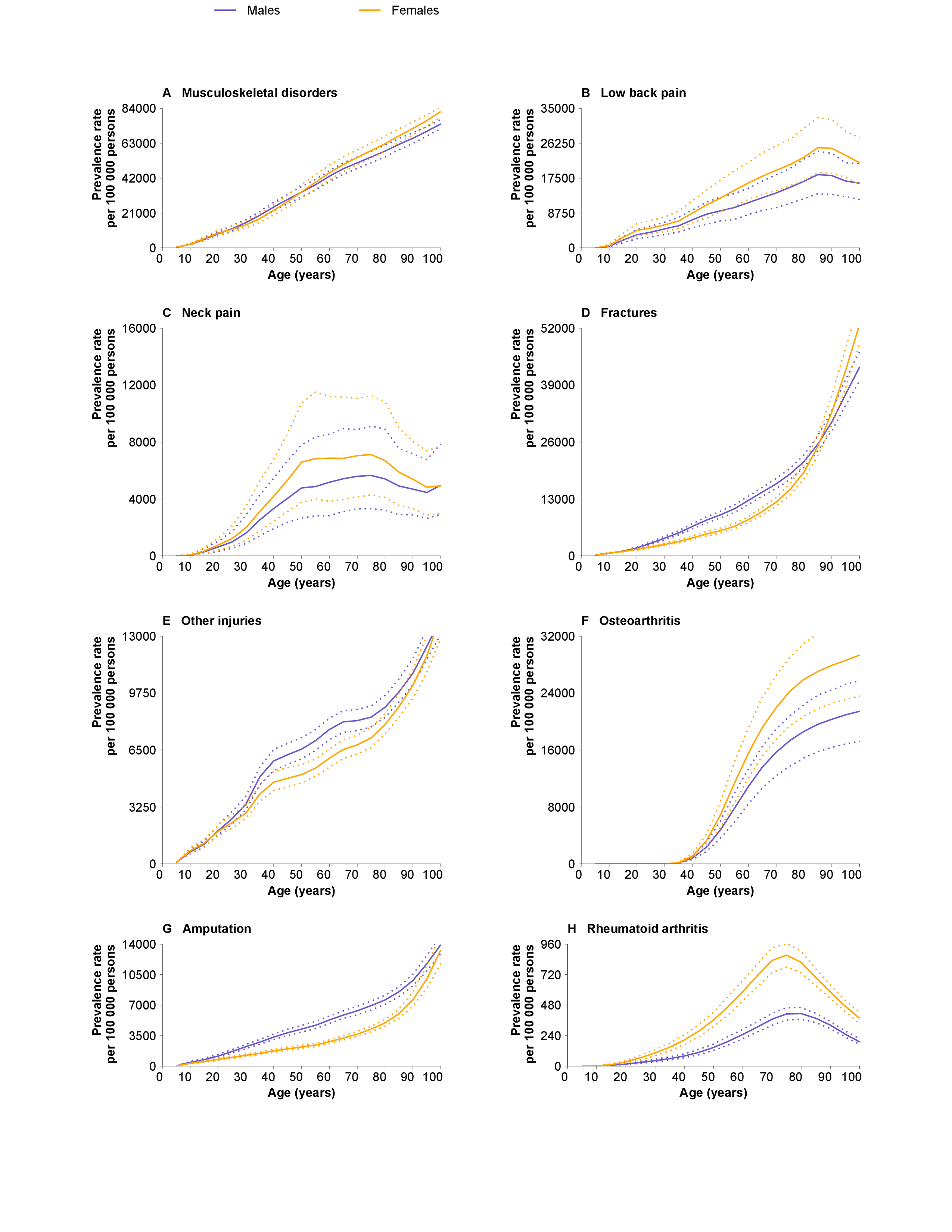

Supplement: Supplementary file 2 [file Data_Sheet_1.zip › Supplementary Figure 7.TIFF]

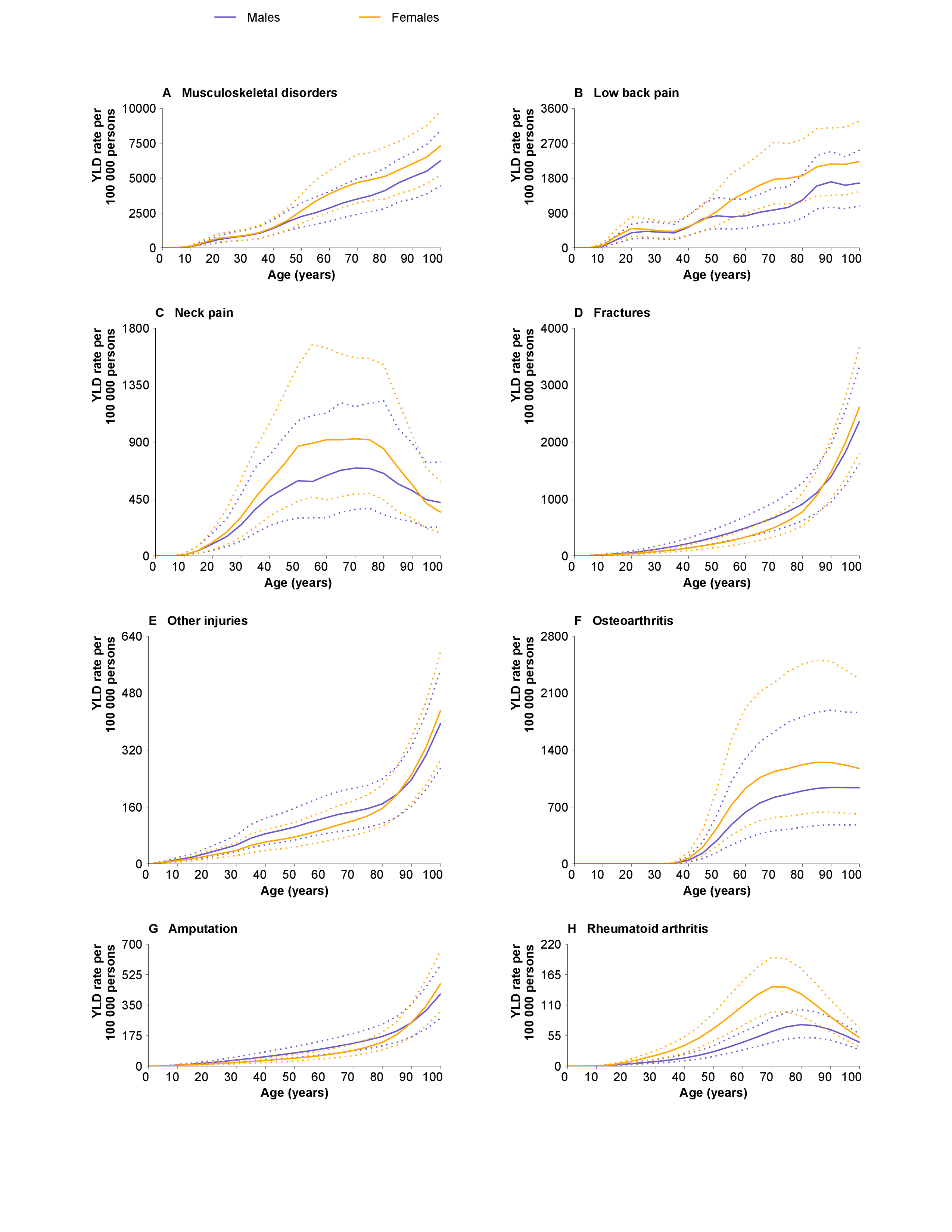

Supplement: Supplementary file 2 [file Data_Sheet_1.zip › Supplementary Figure 8.TIFF]

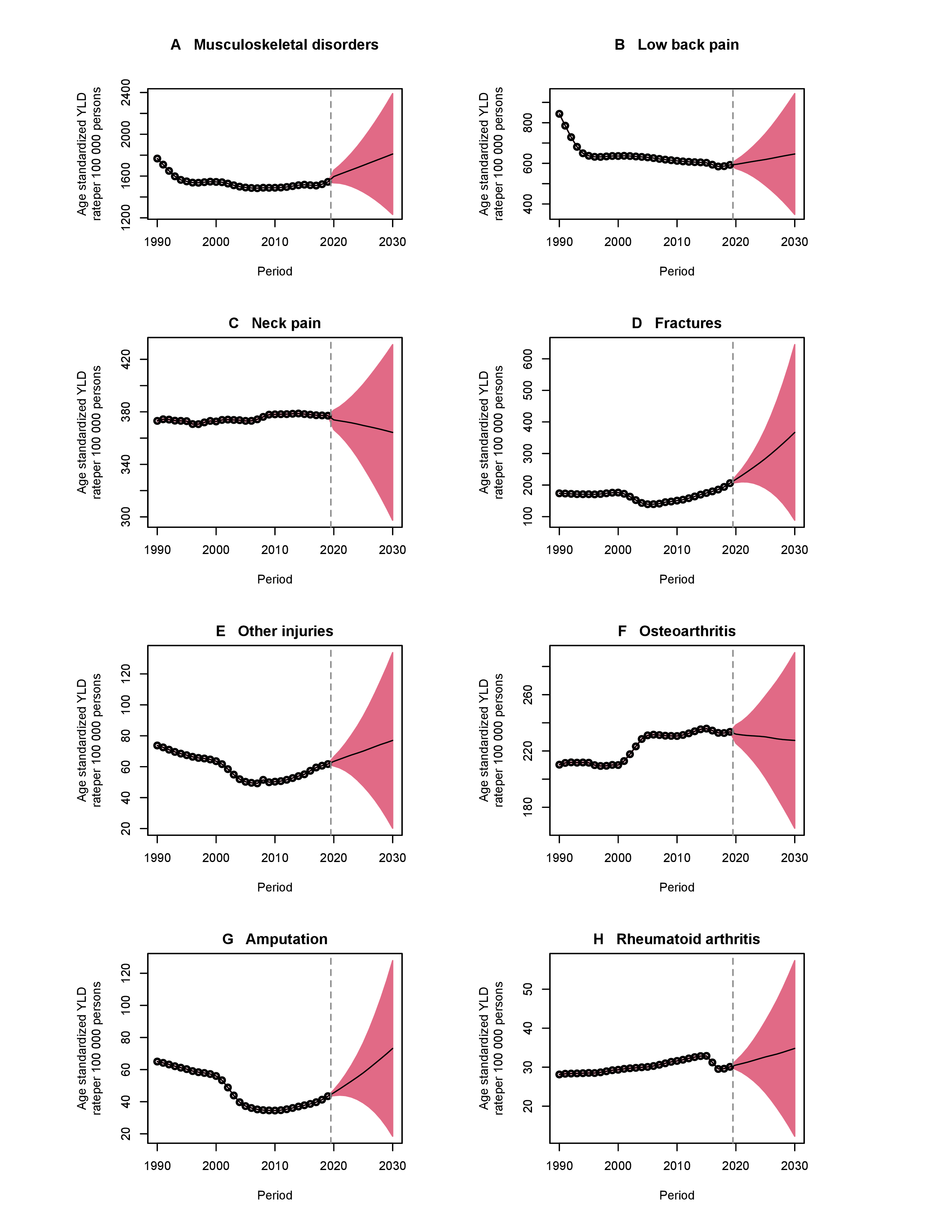

Supplement: Supplementary file 2 [file Data_Sheet_1.zip › Supplementary Figure 9.TIFF]
